# Neuropeptidomes of *Tenebrio molitor* L. and *Zophobas atratus* Fab. (Coleoptera, Polyphaga: Tenebrionidae)

**DOI:** 10.1021/acs.jproteome.1c00694

**Published:** 2022-09-15

**Authors:** Paweł Marciniak, Joanna Pacholska-Bogalska, Lapo Ragionieri

**Affiliations:** † Department of Animal Physiology and Developmental Biology, Institute of Experimental Biology, Faculty of Biology, Adam Mickiewicz University, Poznań 61-614, Poland; ‡ Department for Biology, Institute of Zoology, University of Cologne, Cologne 50674, Germany

**Keywords:** mass spectrometry, peptidomics, transcriptome, differential processing, Coleoptera

## Abstract

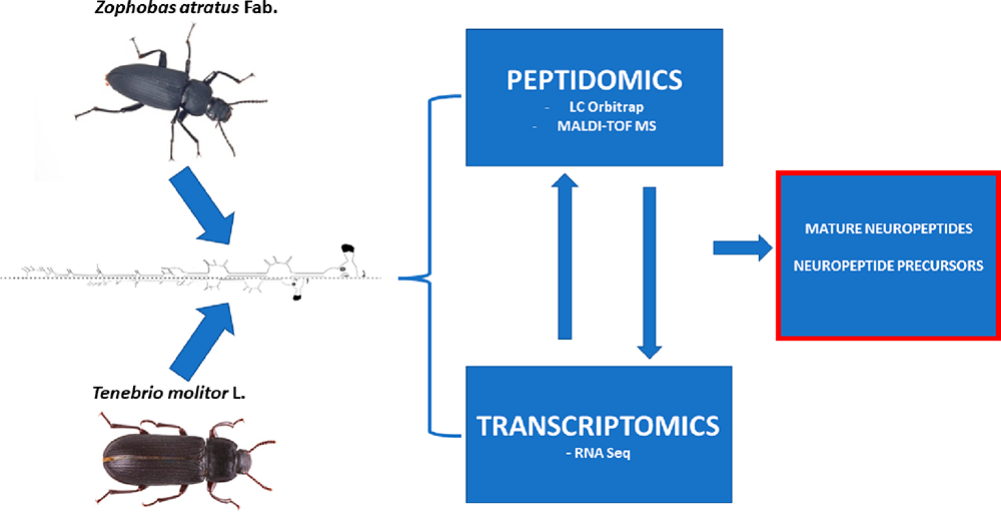

Neuropeptides are signaling molecules that regulate almost all physiological processes in animals. Around 50 different genes for neuropeptides have been described in insects. In *Coleoptera*, which is the largest insect order based on numbers of described species, knowledge about neuropeptides and protein hormones is still limited to a few species. Here, we analyze the neuropeptidomes of two closely related tenebrionid beetles: *Tenebrio molitor* and *Zophobas atratus*—both of which are model species in physiological and pharmacological research. We combined transcriptomic and mass spectrometry analyses of the central nervous system to identify neuropeptides and neuropeptide-like and protein hormones. Several precursors were identified in *T. molitor* and *Z. atratus*, of which 50 and 40, respectively, were confirmed by mass spectrometry. This study provides the basis for further functional studies of neuropeptides as well as for the design of environmentally friendly and species-specific peptidomimetics to be used as biopesticides. Furthermore, since *T. molitor* has become accepted by the European Food Safety Authority as a novel food, a deeper knowledge of the neuropeptidome of this species will prove useful for optimizing production programs at an industrial scale.

## Introduction

Neuropeptides are among the most ancient and diverse signaling molecules in metazoans, regulating several physiological functions, such as behavior, development, and environmental stress response.^1−5^ Endogenous peptides may act both as hormones in the circulatory system and neuromodulators in the peripheral and central nervous systems.^6^ Neuropeptides exert their functions mostly by activating specific cell membrane receptors, such as G-protein-coupled receptors (GPCRs).^7^ For these reasons, neuropeptides and their receptors co-evolved and show conserved evolutionary features.^8^ Neuropeptides are mostly produced in neurons, interneurons, and neurosecretory cells within the central nervous system (CNS),^6,9^ in the peripheral nervous system, and in the endocrine cells of the intestine.^6^ After prohormone processing, a single or multiple copies (paracopies) of mature peptides are released across axonal pathways in the circulatory system and in the CNS or stored and released from neurohemal organs. The majority of paracopies produced from a single neuropeptide precursor share a similar or identical C-terminus and activate the same receptor, while in a few cases, the mature products of a neuropeptide precursor may activate different GPCRs. The latter is the case for genes such as *capa* and *pk/pban (pyrokinin/pheromone biosynthesis activating neuropeptide)*, as demonstrated in *Drosophila melanogaster*.^10−12^

The identification of neuropeptide precursors has accelerated during the last decade thanks to the availability of genomic and transcriptomic data.^13^ In insects, there are currently around 50 recognized genes encoding for neuropeptides, putative neuropeptides, and protein hormones (for a review, see Nässel and Zandawala^14^). Using a combination of transcriptomic and peptidomic analyses, it is possible to simultaneously identify expressed neuropeptide genes and examine the processing of the respective putative bioactive neuropeptides in several species.^7,15−19^ Until recently, most neuropeptide genes and precursors had been described in hemimetabolan insect orders, such as Blattodea, Orthoptera, and Hemiptera, and in a few holometabolan orders, such as Lepidoptera, Diptera, and Coleoptera.^20^ The almost complete set of predicted neuropeptide precursors in Coleoptera was recently described *in silico* by two comparative studies based on publicly available databases.^8,21^ Detailed neuropeptidomes have been studied using mass spectrometry in the large pine weevil *Hylobius abietis* (Polyphaga: Curculionidae)^22^ and in ground beetles of the genus *Carabus* (Adephaga: Carabidae).^18^ One of the main findings of these studies was the loss of several neuropeptide genes, especially in Polyphaga species, commonly found in other insect groups, such as insect *kinins* (only present in Adephaga), *allatostatin a*, *corazonin*, and *allatostatin ccc*.^8,18,22−24^

Many phytophagous Coleoptera species are recognized worldwide as pests capable of causing serious losses both in cultivated and forest areas. Over the years, there has been an increasing use of insecticides^25^ to reduce damage caused by pests.^25^ Besides the negative environmental effects of broad-spectrum insecticides on beneficial species (e.g., pollinators and natural predators of pests), beetles are known to develop resistance against insecticides and xenobiotics (www.pesticideresistance.org). During the last decade, neuropeptide mimetic analogues have been proposed as candidates for the development of species-specific insecticides that target the key physiological functions of the pests only, without harming beneficial species.^18,22,26−28^ In order to apply this strategy, it is necessary to obtain comprehensive knowledge of the neuropeptidomes of both the target pest species (or group of species) and the beneficial species to be protected.^18^ Until now, the insecticidal effects of several neuropeptide analogues were successfully tested in aphids, in moths, and in *T. castaneum*.^29−34^ Currently, the main limitations of this approach are the stability of peptides against endogenous peptidases and the penetration through the gut or cuticle into the hemolymph.^35^

Beetle species belonging to the Tenebrionidae family are commonly employed as models in life science research, such as in medicine, physiology, and agronomy.^25^*T. castaneum* is widely used in genetics, immunology, and developmental biology,^36−38^ but it is not the best candidate for physiological bioassays due to its small size. Alternative beetle model organisms for physiological experiments are the larger *Tenebrio molitor* and *Zophobas atratus*, which have been successfully used for studying tissue- or organ-specific effects of different biologically active molecules, such as neurohormones or alkaloids, as well as in pharmacological studies.^25,39−42^*T. molitor* is currently one of the most cited Coleoptera species on PubMed, with more than 300 publications during the past three years, partly due to the fact that this species was recently accepted by the European Food Safety Authority as a novel food source.^43^

Until now, peptidomic studies have been limited in *T. molitor* and *Z. atratus*. In *T. molitor*, the peptidome of the cerebral ganglia and retrocerebral complex was investigated with matrix-assisted laser desorption ionization time-of-flight (MALDI-TOF)^44^ using the deduced neuropeptide precursor sequences of *T. castaneum* to identify evolutionarily conserved neuropeptides between these two tenebrionid species. Four neuropeptides from the cerebral ganglia and corpora cardiaca-corpora allata (CC-CA) were reported with this approach.^44^ In *Z. atratus*, nine neuropeptides were identified using a similar approach.^45^ More recently, the products and distribution of *capa* and *pk* genes in the CNS of *Z. atratus* were investigated using MALDI-TOF^46^ and the almost complete set of predicted neuropeptide precursors of *T. molitor* was included in a comparative study among Coleoptera.^8^ The aim of the present study was to identify neuropeptides and neuropeptide-like and protein hormones and their distribution in the nervous systems of *T. molitor* and *Z. atratus*. The information obtained will expand our knowledge of processed neuropeptides in beetles and will be useful for the design of peptide mimetic analogues to be tested as biopesticides. At the same time, increased knowledge of *T. molitor* neuropeptidomes could turn out to be useful for optimizing the production of larvae at an industrial scale. To achieve this goal, we combined the information obtained by transcriptome and mass spectrometry analyses of the nervous systems of *T. molitor* and *Z. atratus*. We also took advantage of recently available *T. molitor* genome assemblies^47,48^ to solve ambiguities or missing information of our transcriptome. Overall, we identified around 50 neuropeptide precursors in both species, the majority of which confirmed by a combination of two mass spectrometry approaches: one based on MALDI-TOF direct profiling of CNS tissues and the second based on extracts of the complete CNS analyzed with LC coupled to an Orbitrap mass spectrometer.

## Materials and Methods

### Insects and Rearing Conditions

Adults of *T. molitor* (Linnaeus, 1758) and *Z. atratus* (Fabricius, 1776) were obtained from a culture maintained at the Department of Animal Physiology and Developmental Biology, Adam Mickiewicz University, Poznań, Poland. *T. molitor* were reared as described previously,^49^ whereas *Z. atratus*, according to the procedure described in Quennedey et al.^50^

### RNA Extraction

Total RNA was extracted from the brain and retrocerebral complex of 10 adults of each species using TRIzol (Invitrogen, Grand Island, NY, USA), following the manufactory instructions of the Beijing Genomics Institute (BGI, China). RNA quality parameters were estimated using RNA concentration (ng/μL) and the RNA integrity number as implemented in an Agilent 2100 Bioanalyzer.

### Library Construction and Transcriptome Sequencing

Libraries were sequenced using an Illumina Truseq RNA sample preparation kit (Illumina, San Diego, USA) at BGI, as already described in ref (51). Briefly, once total RNA was extracted, magnetic beads with Oligo (dT) were used to isolate mRNA and mixed with buffer to fragment the mRNA into short fragments. The cDNA was synthesized using these mRNA fragments as templates. First-strand cDNA was generated using random hexamer-primed reverse transcription, followed by second-strand cDNA synthesis, double-stranded cDNA purification and resolution using elution buffer for end reparation, and Poly (A) tail addition. Finally, after ligating the Illumina sequencing paired end (PE) adaptors, products were purified on Tris–acetate–ethylenediamine tetraacetic acid agarose gel and suitable fragments were selected for PCR amplification in order to enrich the purified cDNA template. Quality control of the cDNA library was performed using an Agilent 2100 Bioanalyzer. Finally, the resulting cDNA library was sequenced using an Illumina HiSeqTM4000 with strategies of 100 bp PE at BGI.

### De Novo Assembly of Nucleotide Sequences and Quality Control

Raw data were filtered by removing adapters and low quality reads at BGI. The resulting filtered RAW reads were submitted to NCBI (Sequence Read Archives (SRA): SRR11184806 and SRR11358229, BioProject PRJNA608239 for *T. molitor* and SRR11178058 and SRR11178059, BioProject PRJNA608269 for *Z. atratus*). Filtered reads were de novo assembled by means of Trinity 2.2.0, using the trinity read normalization option. Trinity transcriptome assemblies were submitted to the NCBI Transcriptome Shotgun Assembly (TSA) database. The *T. molitor* assembly has been deposited at DDBJ/EMBL/GenBank under accession number GIPG00000000. The version described in this paper is the first version, GIPG01000000. The *Z. atratus* assembly has been deposited at DDBJ/EMBL/GenBank under accession number GIPJ00000000. The version described in this paper is the first version, GIPJ01000000. In addition to our transcriptome data, we also used the recently published genome of *T. molitor* (BioProject: PRJNA579236; BioProject: PRJEB44755)^47,48^ to complete neuropeptide ambiguities or missing neuropeptide precursors.

### Compiling of Precursor Sequences

For identification of precursor sequences, we performed a search by homology (tBLASTn) using sequences of known insect neuropeptide precursors as reference queries,^8,16,18,22,52^ on a local computer as implemented in BLAST+.^53^ Positive hits within the transcriptome assembly were translated into proteins using the ExPASy translate tool^54^ (web.expasy.org/translate/). Signal peptides for the putative precursors were predicted using the SignalP 5.0 and 6.0 servers^55^ (www.cbs.dtu.dk/services/SignalP/). Cleavage sites were initially manually assigned based on known cleavage sites in homologous precursors from other species. Missing neuropeptide precursors were also searched in the raw data using the BLAST+ algorithm.

### Tissue Preparation for MALDI-TOF MS

Animals were kept at −4 °C for 15 min before dissecting the CNS in insect saline solution (NaCl 126 mM, KCl 5.4 mM, NaH_2_PO_4_ 0.17 mM, KH_2_PO_4_ 0.22 mM; pH 7.4).^56^ We carefully dissected the antennal lobe, the neurohemal organs from the abdominal and thoracic ganglia and the retrocerebral complex and the frontal and terminal ganglia as previously described.^18^

### Tissue Preparation for Orbitrap MS

Dissected tissues were briefly washed in a drop of distilled water to remove salt contaminations and immediately transferred to a 30 μL extraction solution (Buffer 1: 90% methanol, 1% formic acid [FA]). Tissues were dissociated in an ultrasonic bath (Transonic 660/H, Elma Schmidbauer GmbH, Hechingen, Germany) at 4 °C for 5 min and with an ultrasonic probe three times for 2 s (Bandelin Sonopuls HD 200, Bandelin electronic GmbH, Berlin, Germany), respectively. Extracts were then centrifuged for 20 min at 15,000 rpm at 4 °C. Supernatants were transferred to a fresh tube (Eppendorf, Hamburg, Germany), and methanol was evaporated in a vacuum concentrator (Hetovac VR-1, Heto Lab Equipment, Roskilde, Denmark).

### Reduction of Disulfide Bonds, Carbamidomethylation of Cysteines, and Protein Digestion

To analyze protein hormones we used a fraction of the extracts previously prepared as described in ref (18). Protein extracts were quantified using a Direct Detect infrared spectrometer (Merck KGaA, Darmstadt, Germany) for subsequent protein digestion. We used 10 μg of proteins for each sample. Extracts were subjected to disulfide reduction by adding dithiothreitol (DTT) to a final concentration of 20 mM at 37 °C for 1 h. Subsequently, samples were immediately subjected to carbamidomethylation by adding iodoacetamide to a final concentration of 40 mM for 30 min in darkness. The reaction was quenched by adding DTT at a final concentration of 40 mM. For protein digestion, we first added endoproteinase Lys-C (Lys-C; Wako, Richmond, VA, U.S.A) at 0.5 μg/μL for 4 h at 37 °C, followed by trypsin at 1 μg/μL (Sigma-Aldrich, Steinheim, Germany), and incubated it at 37 °C for 12 h. Both enzymes were added at an enzyme/substrate ratio of 1:75 following the manufacturer’s instructions. Enzymatic digestion was stopped by adding FA to a final concentration of 1%.

### Quadrupole Orbitrap MS

Before being injected into the nanoLC system, all samples were desalted using self-packed Stage Tip SDB-RPS (IVA Analysentechnik e. K., Meerbusch, Germany) spin columns as described previously.^57^ Peptides were first separated with an EASY nanoLC1000 UPLC system (Thermo Fisher Scientific) using in-house packed 50 cm RPC18 columns (fused Silica tube with ID 50 μm ± 3 μm, OD 150 μm ± 6 μm; Reprosil 1.9 μm, pore diameter 60 Å; Dr. Maisch GmbH, Ammerbuch-Entringen, Germany) and a binary buffer system (A: 0.1% FA; B: 80% ACN, 0.1% FA). The UPLC was coupled to a Q-Exactive Plus (Thermo Fisher Scientific) mass spectrometer. Running conditions were as described in ref (56). Mass spectrometry proteomics data were deposited at the ProteomeXchange Consortium via the PRIDE^58^ partner repository under dataset identifiers PXD032947 (doi:10.6019/PXD032947) for *T. molitor* and PXD033000 (doi:10.6019/PXD033000) for *Z. atratus*.

Raw data were analyzed using PEAKS Studio 10 (BSI, ON, Canada).^59^ Samples not subjected to enzymatic digestion were matched against an internal database containing the transcriptome-derived precursor sequences of *T. molitor* and Z. atratus, as well as the six frame translation of the complete transcriptomes. For analyses with PEAKS, the same set of post-translational modifications was used, and peptides were searched against the same internal databases with a parent error mass tolerance of 10 ppm and fragment mass error tolerance of 0.05 Da. No enzyme mode was selected. Variable post-translational modifications included in the searches were oxidation at methionine, acetylation at the N-terminus, amidation of C-terminal glycine, pyroglutamate from glutamine, pyroglutamate from glutamic acid, and disulfide bridges. The false discovery rate (FDR) was determined by a decoy database search implemented in PEAKS 10 and set below 1%. To provide the accurate monoisotopic mass of a peptide, Q Exactive Orbitrap RAW data were corrected prior to the analysis (precursor mass correction only). Fragment spectra with a peptide score (−10lg*P*) equivalent to a *P*-value of about 1% were manually reviewed. Peptide spectrum matches with an FDR of 0.1% (approximately −10log*P* values higher than 30) were subsequently manually checked. Samples that were enzymatically treated with LysC and trypsin were analyzed using the same parameters but with enzyme mode set to trypsin and carboxymethylation as a fixed modification.

### MALDI-TOF MS

For direct tissue profiling, small parts of the nervous system, including the antennal lobe and neurohemal organs, were carefully dissected, washed in a drop of distilled water, transferred on a MALDI-TOF sample plate, and allowed to air-dry. Dried tissues were covered with 0.3–0.4 μL drops of matrix. Matrices used in this study were 2,5-dihydroxybenzoic acid (DHB, Sigma-Aldrich) at a final concentration of 10 mg/mL in 1% aqueous FA containing 20% acetonitrile (ACN) (v/v) and α-cyano-4-hydroxycinnamic acid (α-CHCA Sigma- Aldrich) at 10 mg/mL in 60% ethanol, 36% ACN, 4% water (stock solution) and dissolved in 50% methanol/water (2:1 50% methanol/CHCA stock solution). Mass fingerprint (MS^1^) and ion fragmentation (LIFT mode – MS^2^) spectra were acquired using two instruments: an UltrafleXtreme MALDI-TOF mass spectrometer (Bruker Daltonik GmbH, Bremen, Germany) and a MALDI TOF ABI 4800 Proteomics Analyzer (Applied Biosystems Framingham, MA). The UltrafleXtreme was mostly used for MS^1^ under manual control in reflectron-positive ion mode and for MS^2^ experiments with LIFT technology without CID for both DHB- and α-CHCA-coated samples, while the MALDI TOF ABI 4800 was used exclusively for MS^2^ in gas off mode with α-CHCA-coated samples. MS^2^ spectra were manually reviewed by comparing with theoretical ion fragments (prospector.ucsf.edu/prospector/mshome.htm). For external calibration, a mixture of proctolin, *Drosophila melanogaster* short neuropeptide 2^12−19^ (sNPF-2^12−19^), *Periplaneta americana* (Pea-FMRFa-12), *Locusta migratoria* periviscerokinin (Lom-PVK), Pea-SKN, and glucagon for the lower mass range (*m/z* 600–4000) and a mixture of bovine insulin, glucagon, and ubiquitin for the higher mass range (*m/z* 3000–10,000) were used. Spectra were analyzed using flexAnalysis 3.4 (Bruker Daltonik) and Data Explorer v. 4.3 (Applied Biosystems). MALDI raw data were deposited at the Zenodo (doi: 10.5281/zenodo.6913565) for *T. molitor* and PXD033000 (doi: 10.5281/zenodo.6948001) for *Z. atratus*.

## Results and Discussion

### Transcriptome Identifications

The numbers of identified neuropeptide, neuropeptide-like, and protein hormone precursors in *T. molitor* and *Z. atratus* were 60 and 59, respectively (Table 1). In the *T. molitor* transcriptome, we found several splice forms for CAPA, neuropeptide F1 (NPF1), orcokinin-like (Ork-like), agatoxin-like peptide (ALP), ion transport peptide (ITP), arthropod insulin-like growth factor (aIGF_a-b_),^52^ and corticotropin-releasing factor-like diuretic hormone CRF-DH (Table 1; Figures S1 and S2). Moreover, we found a few precursors that did not match the ones described in Veenstra.^8^ Most differences arose in proctolin, orcokinin, prothoracicotropic hormone (PTTH), and calcitonin-like diuretic hormone (CT-DH) precursors. We assembled two *proctolin* genes, which have been fully confirmed in the *T. molitor* genome. The second gene (*proctolin 2*) differs considerably at the precursor N-terminal from the one described in Veesntra.^8^ In the genome of *T. molitor*, we also found a second allele for *proctolin 2* (Figure S3). Orcokinin and orcomyotropin peptides were originally described in Crustacea,^60,61^ and two *orcokinin* genes were later described in *Daphnia*.^62^ Insect orcokinins are encoded by a single gene with two splice variants (a and b).^63^ In tenebrionids, the pre-propeptides of orcokinin transcripts a and b do not contain any sequence with a typical orcokinin (NXDEIDR) or orcomyotropin (FDAFTTGF) motif.^64^ We therefore named the two splice variants orcokinin-like transcripts a and b (OK-like_a_ and OK-like_b_). In transcript a, we identified two splice forms (named OK-like_a1_ and OK-like_a2_; Figure S2A), both of which contain a different signal peptide from the one described in Veenstra.^8^ In our transcriptome, we could detect only a small fragment of the OK-like_b_ precursor containing four peptides (SLDGIGGGNLV-NH_2_; SLDRIGGGNLV-NH_2_; STDGIDGDLI-NH_2_; and SLARTNKLN-NH_2_). In order to complete the OK-like_b_ precursor, we combined genomic and transcriptomic data and found at least 30 amidated peptides similar to those described in *T. castaneum* (Figure S2B). The PTTH-like precursor described in Veenstra^8^ contains several gaps in the central region of the precursor. Finally, Veenstra^8^ described two additional splice forms of the calcitonin-like diuretic hormone, but we did not include them in our precursor list as they do not encode any CT-DH.

**Table 1:** Precursors for Neuropeptides and Neuropeptide-Like and Protein Hormones Identified in the Transcriptomes of *T. molitor* and *Z. atratus*^a^

		*T. molitor*			*Z. atratus*		
designation	abbreviations	accession	amino acids	MS	accession	amino acids	MS
**neuropeptides**							
adipokinetic hormone 1	AKH	ON086791	72	+	ON155921	71	+
adipokinetic hormone/corazonin-related peptide	ACP	ON086792	82	+	ON155922	88	+
allatostatin C	AstC	ON086793	103	+	ON155923	104	+
allatostatin CC	AstCC	ON086794	135	+	ON155924	134	+
allatotropin	AT	ON086795	101	+	ON155925	104	+
antidiuretic factor b-1	AFB-1	ON110494	137	+	ON155926	129	+
antidiuretic factor b-2	AFB-2	–	–	–	ON155927	165	+
antidiuretic factor b-3	AFB-3	–	–	–	ON155928	114	–
antidiuretic factor b-4	AFB-4	–	–	–	ON155929	144	–
calcitonin 1	calcitonin 1	ON110495	120	–	ON155930	121	–
calcitonin 2	calcitonin 2	ON110496	115^b^	–	–	–	–
calcitonin-like diuretic hormone	CT-DH	ON110497	119	+	ON155931	119	+
CAPA transcript a	CAPA_a_	ON110498	174	+	ON155932	154	+
CAPA transcript b	CAPA_b_	ON110499	158	+	–	–	–
CCHamide1	CCHa-1	ON110500	159	+	ON155933	155	+
CCHamide2	CCHa-2	ON110501	113	+	ON155934	112	+
CNMamide	CNMa	ON110502	141	–	ON155935	95^c^	–
corticotropin-releasing factor-like diuretic hormone (DH37)	CRF-DH37	ON110503	125	+	ON155936	128	+
corticotropin-releasing factor-like diuretic hormone (DH47)	CRF-DH47	ON110504	154	+	ON155937	157	+
crustacean cardioactive peptide	CCAP	ON110505	143	+	ON155938	143	+
ecdysis triggering hormone	ETH	ON110506	139	–	ON155939	77^c^	–
elevenin	elevenin	ON110507	126	+	ON155940	83^c^	–
FMRFamide-related peptides	FMRF	ON110508	207	+	ON155941	200	+
HanSolin	HanSolin	ON110509	121	+	ON155942	117	–
IDL-containing	IDL	ON110510	204	+	ON155943	204	+
inotocin (vasopressin-like)	inotocin	ON110511	151	+	ON155944	152	+
insect parathyroid hormone	IPH	ON110512	110	+	ON155945	110	–
myoinhibitory peptide	MIP	ON110513	187	+	ON155946	185	+
myosuppressin	MS	ON110514	91	+	ON155947	102	+
natalisin	natalisin	ON110515	163	+	ON155948	165	+
neuropeptide F1_a_	NPF1_a_	ON110516	85	+	ON155949	82	–
neuropeptide F1_b_	NPF1_b_	ON110517	123	–	ON155950	120	–
neuropeptide F2	NPF2	ON110518	90	+	ON155951	89	+
orcokinin-like transcript a1	OK-like_a1_	ON155962	171	+	ON155952	170	+
orcokinin-like transcript a2	OK-like_a2_	ON155961	146	–	–	–	–
orcokinin-like transcript b	OK-like_b_	ON155963	439^b^	–	–	–	–
pigment dispersing factor	PDF	ON110519	99	+	ON155953	100	+
proctolin 1	proctolin 1	ON155964	83	+	ON155954	83	+
proctolin 2 allele 1	proctolin 2_1_	ON155965	77	+	–	–	–
proctolin 2 allele 2	proctolin 2_2_	ON155966	77^b^	+	–	–	–
pyrokinin	PK	ON110520	163	+	ON155955	168^c^	+
RFLamide	RFLa	ON110521	185	–	ON155956	185	–
RYamide	RYa	ON110522	128	+	ON155957	130	+
short neuropeptide F	sNPF	ON125379	97	+	ON155958	100	+
SIFamide	SIFa	ON125380	75	+	ON155959	75	+
sulfakinin	SK	ON125381	116	+	ON155960	115	+
tachykinin-related peptide	TKRP	ON125382	272	+	ON155969	273	+
trissin	trissin	ON125383	100	–	ON155970	99	–
**neuropeptide-like**							
agatoxin-like peptide a	ALP_a_	ON125384	108	+	ON155971	108	+
agatoxin-like peptide b	ALP_b_	ON125385	99	+	ON155972	99	+
neuropeptide-like precursor 1	NPLP1	ON125386	423	+	ON155973	422	+
NVP-like	NVP	ON125387	322	+	ON155974	317	+
Periplaneta neuropeptide-like precursor	Pea-NPLP	ON125388	691	+	ON155975	679	+
**protein hormones**							
bursicon alpha	Burs-α	ON155991	160^b^	–	ON155976	167	–
bursicon beta	Burs-ß	ON155992	135	+	ON155977	133^c^	–
eclosion hormone 1	EH 1	ON125389	81	–	ON155978	82	–
eclosion hormone 2	EH 2	ON125390	77	–	ON155979	77	–
glycoprotein hormone alpha 2	GPA2	ON125391	122	+	ON155980	122	+
glycoprotein hormone beta 5	GPB5	ON125392	155	+	ON155981	155	+
arthropod insulin-like growth factor_a_	aIGF	ON155967	162	–	–	–	–
arthropod insulin-like growth factor_b_	aIGF	ON125395	162	–	ON155984	179	–
insulin-like peptide 1	ILP 1	ON125393	125	+	ON155982	125	+
insulin-like peptide 2	ILP 2	ON125394	133	–	ON155983	125	–
insulin-like peptide 3	ILP 3	ON155968	121^c^	+	–	–	–
insulin-like peptide 4 (allele 1)	ILP 4_1_	ON125396	104	+	–	–	–
insulin-like peptide 4 (allele 2)	ILP 4_2_	ON125397	104	+	–	–	–
relaxin	relaxin	ON125398	145	–	ON155985	83^c^	–
ion transport peptide-like_a_	ITP_a_	ON125399	136	+	ON155986	136	+
ion transport peptide-like_b_	ITP_b_	ON125400	120	+	ON155987	120	–
ITG-like	ITG	ON125401	214	+	ON155988	214	+
neuroparsin	neuroparsin	ON125402	107	+	ON155989	109	+
prothoracicotropic hormone	PTTH	ON125403	179^c^	–	ON155990	184	+

a Sequences are listed in the Supporting Information S1. Different transcripts are marked with subscript characters and alleles with subscript numbers.

b Completed with BioProject: PRJNA579236; BioProject: PRJEB44755PRJNA646689.

c Incomplete.

Our precursor list also includes five additional precursors known across insects: the antidiuretic factor ADF;^65^ IDL-like; neuropeptide F1;^66^ and two recently described precursors, the insect parathyroid hormone (iPTH)^67^ and *Periplaneta* neuropeptide-like (Pea-NPLP = PaOGS36577).^68^ The latter is homologous to a precursor described in the ant *Cataglyphis nodus* with the name of Fliktin.^69^ Finally, we detected an additional precursor of insulin-like peptide (ILP) in the *T. molitor* transcriptome (Table1; Supporting Information S1).

In *Z. atratus*, we identified only three splice forms in *alp*, *npf1*, and *itp* (Figure S4). We could not find any sequence for *OK-like_b_* and *calcitonin 2*, most likely because these two genes are expressed in the midgut tissue, which was not covered in our transcriptome.^64,70^

Based on the investigated transcriptomes and genomes, we further confirmed the absence of *allatostatin a*, *corazonin*, *insect kinins*, and *allatostatin ccc* genes in tenebrionids. Moreover, we could not identify the recently described genes for *smyamide*, a paralog of *sifamide*,^71^*gonadulin*,^72^ and *carausius-like neuropeptide*.^16,19^

### Mass Spectrometry Identifications

Overall, we confirmed the presence of 50 and 40 neuropeptide precursor products and transcripts across the CNS of *T. molitor* and *Z. atratus*, respectively (Table 1). The neuropeptide products that were not detected in either species using mass spectrometry were calcitonins 1 and 2, CNM, ecdysis triggering hormone (ETH), OK-like_b_, RFLa, and trissin. Calcitonins and OK-like_b_ are mostly expressed in the midgut and could be less abundant in our preparations.^63,64,70^ Of the protein hormones, EH, relaxin, ILP-2, and aIGF_a-b_ were not detected in either species. EH and ETH have key functions in regulating ecdysis behavior in insects and therefore were likely not expressed in the CNS of larvae and adults.^73^ In *Z. atratus*, we could not confirm ADF-b3, ADF-b4, HanSolin, iPTH, NPF1, and RYa precursors.

The two mass spectrometry approaches yielded similar results except for inotocin, NPF-1, and NPF-2, which were detected only in the MALDI-TOF fingerprints, and ADF, HanSolin, iPTH, natalisin, and OK-like_a_, which were confirmed only by Orbitrap analyses in *T. molitor*. Similarly, in *Z. atratus*, Orbitrap analyses could detect two ADF peptides and CCHa_2_, whereas in MALDI-TOF, we confirmed ALP, CRF-DH37, crustacean cardioactive peptide (CCAP), inotocin, and RYa peptides.

Using protein tryptic digestion, we detected several fragments of neuropeptides and neuropeptide-like precursor peptide (PP) and digested fragments of large protein hormones such as bursicon (Burs-β), glycoprotein hormone alpha 2 (GPA2), glycoprotein hormone beta 5 (GPB5), ITG-like (ITG), and neuroparsin (Supporting Information S1, Tables S1 and S2).

### Neuropeptide Distribution across the CNS

**Figure 1:**
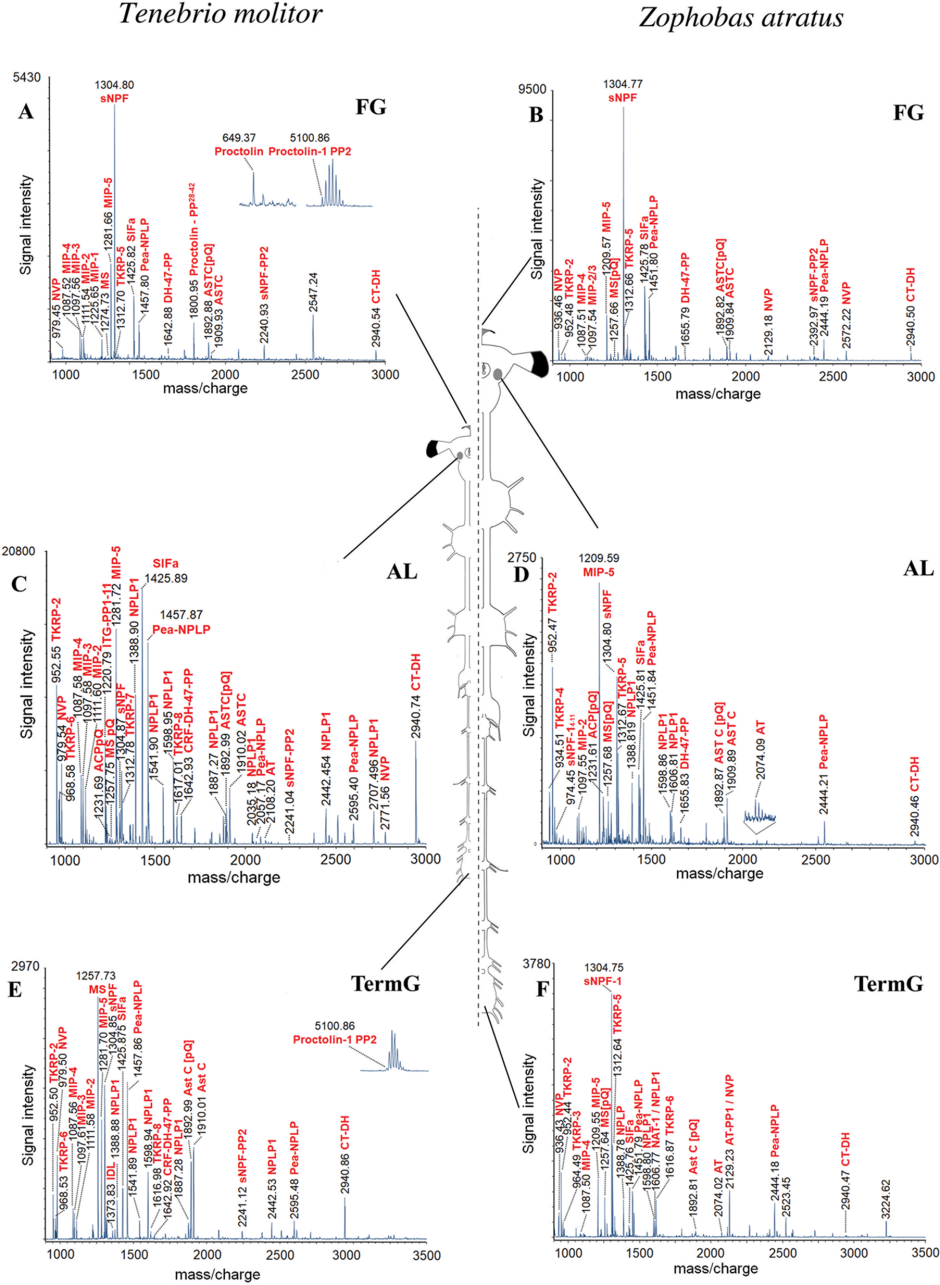
MALDI-TOF MS^1^ spectra obtained by direct tissue profiling of frontal ganglia and neuropile regions of the CNS of *T. molitor* (left panel) and *Z. atratus* (right panel). The mass spectra illustrate a tissue-specific distribution of neuropeptides across the CNS. (A) Mass spectrum of *T. molitor* FG preparation with prominent ion signals for sNPF, SIFa, Proctolin 1, and Pea-NPLP (*m/z* 800–3000). (B) Mass spectrum of *Z. atratus* FG preparation with particularly prominent ion signals for sNPF, SIFa, and Pea-NPLP (*m/z* 800–3000). (C) Mass spectrum of *T. molitor* AL with prominent ion signals for SIFa, ITG, NPLP1, MIP, TKRP, and Pea-NPLP (*m/z* 800–3000). (D) Mass spectrum of *Z. atratus* AL with prominent ion signals for MIP, TKRP, sNPF, and Pea-NPLP (*m/z* 800–3000). (E) Mass spectrum of *T. molitor* terminal ganglion (TermG) preparation with predominant ion signals of MS, sNPF, proctolin, and Pea-NPLP (*m/z* 900–3500). (F) Mass spectrum of *Z. atratus* TermG preparation with predominant ion signals of sNPF and TKRP-5 (*m/z* 900–3500).

**Figure 2:**
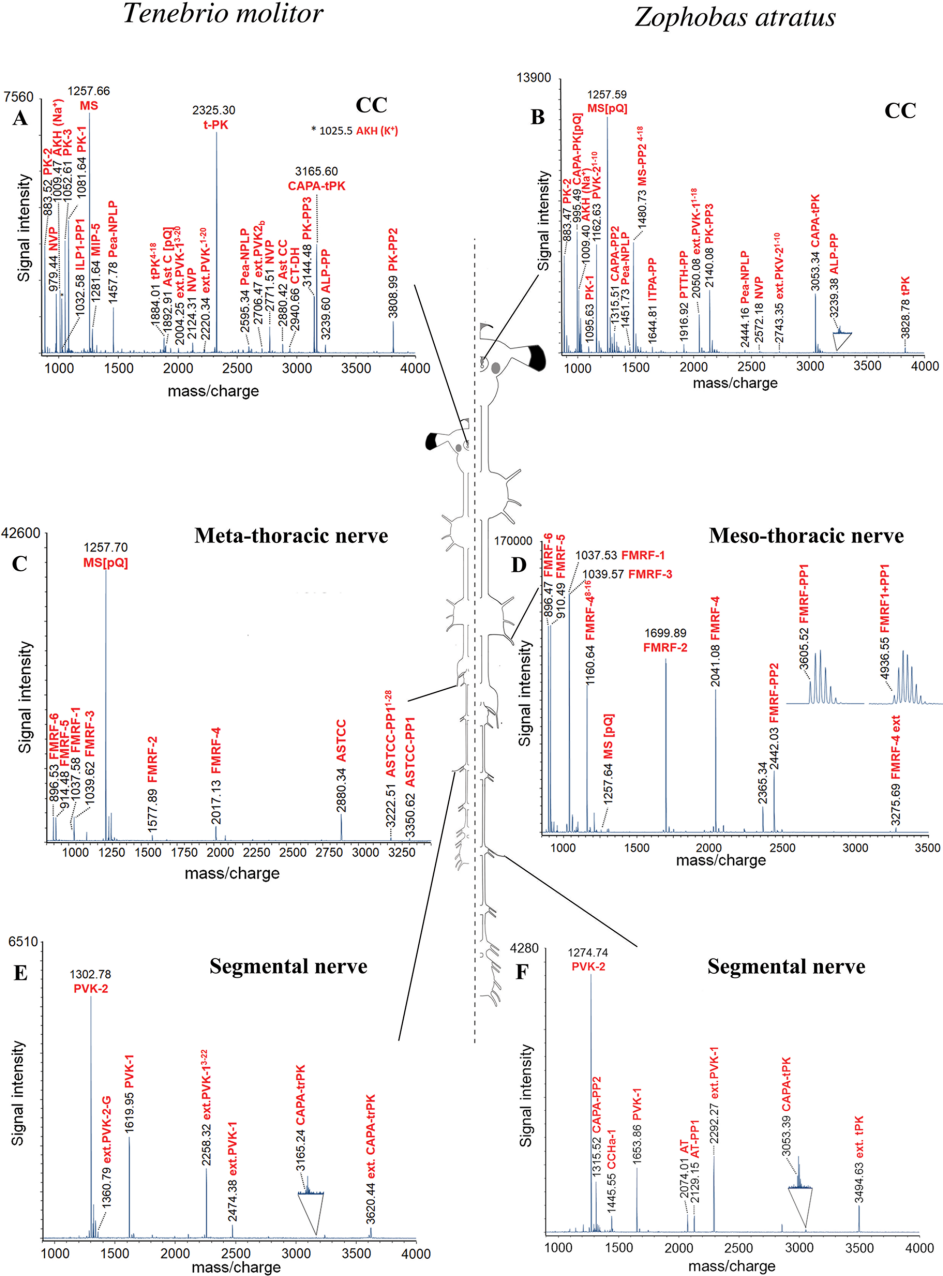
MALDI-TOF MS^1^ spectra obtained by direct tissue profiling of neurohemal organs of *T. molitor* (left panel) and *Z. atratus* (right panel). (A) Mass spectrum of *T. molitor* corpus cardiacum (CC) preparation with prominent ion signals for MS, AKH, PK, and CAPA peptides (*m/z* 800–4000). (B) Mass spectrum of *Z. atratus* CC preparation with prominent ion signals for MS (including the PP MS-PP2^4−18^), AKH, PK, and CAPA peptides (CAPA-PK and tryptoPK), including truncated forms of periviscerokinins (PVK-1 and 2) (*m/z* 800–4000). (C) Mass spectrum of *T. molitor* meta-thoracic SN preparation with predominant ion signal for MS and FMRF peptides. Additionally, ion signals for ASTCC (including the intermediate precursor peptides ASTCC-PP1^1−28^ and ASTCC-PP1) were detected (*m/z* 800–3500). (D) Mass spectrum of *Z. atratus* meso-thoracic SN preparation with ion signals for FMRF (*m/z* 900–3500). (E) Mass spectrum of a *T. molitor* abdominal SN with ion signals of CAPA peptides (*m/z* 900–4000). (F) Mass spectrum of the *Z. atratus* abdominal SN with ion signals of CAPA peptides (*m/z* 900–4000).

Direct tissue profiling offers the possibility to investigate the distribution of neuropeptides in the CNS and the differential processing of neuropeptide precursors.^46^ For these purposes, we analyzed two neuropil regions within the CNS (the antennal lobe and posterior terminal ganglion), the frontal ganglion and the major neurohemal organs of tenebrionids (corpora cardiaca, thoracic segmental nerves, and abdominal segmental nerves). For an overview of neuropeptide distribution, see Figures 1 and 2, Table 2, and S3.

**Table 2:**
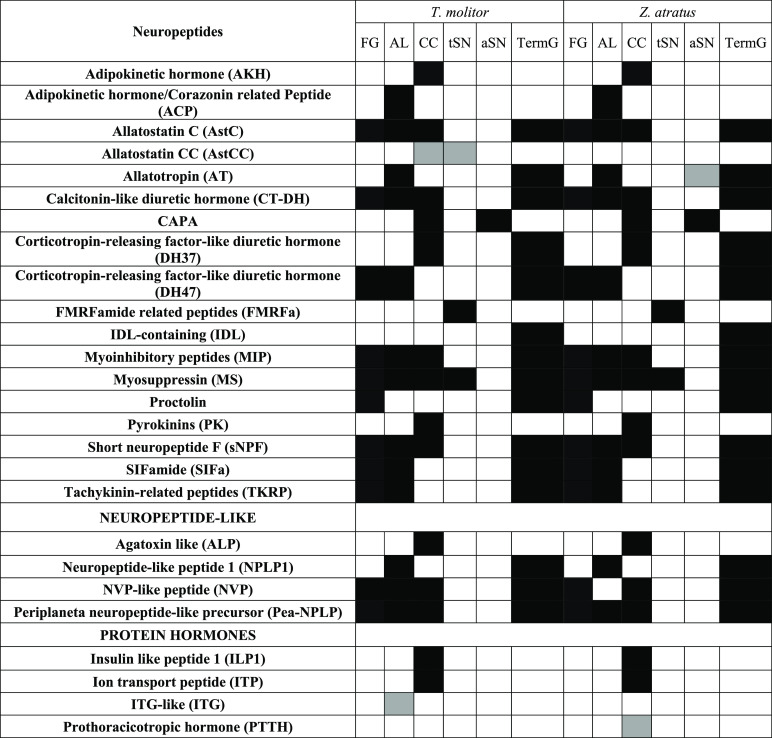
Distribution of Neuropeptide Precursor Products throughout the Nervous Systems of *T. molitor* and *Z. atratus* Detected in MALDI-TOF MS^1^ Spectra^a^

a The presence of a peptide is indicated in black when shared by both species or in gray when not observed in both species. If a neuropeptide is not detected in the listed tissues (i.e., pigment dispersing factor is present only in pars intercerebralis), the precursor is not mentioned in the table. FG, frontal ganglion; AL, antennal lobe; CC, corpora cardiaca; tSN, thoracic segmental nerve; aSN, abdominal segmental nerve; and TermG, terminal ganglia.

The frontal ganglion (FG) is part of the stomatogastric nervous system (SNS) and is involved in the neural regulation of foregut activity.^74^ In beetles, it is also well connected to both the tritocerebrum and the retrocerebral complex (RCC). In both analyzed species, the most abundant neuropeptides in the FG were the short neuropeptides F (sNPF), myoinhibitory peptides (MIP), Pea-NPLP, and SIFamide (SIFa), followed by allatostatin C (AstC), CT-DH, myosuppressin (MS), NVP-like peptide (NVP), proctolin, and tachykinin-related peptides (TKRP) (Figure 1A,B). In addition to the above-mentioned neuropeptides, we also detected a PP of the CRF-DH47 transcript in the FG of both species.

The antennal lobes (AL) are a portion of the deutocerebrum mostly involved in the elaboration of olfactory information and contain several neuropeptides expressed in the cerebral ganglia.^75^ In the AL of *T. molitor*, we could confirm products of the adipokinetic hormone/corazonin-related peptide (ACP), AstC, allatotropin (AT), CRF-DH-47, CT-DH, ITG, NVP, MS, MIP, NPLP1, Pea-NPLP, SIFa, sNPF, and TKRP (Figure 1C), whereas we could not confirm ITG and NVP in the *Z. atratus* AL (Figure 1D), even though they were detected in other regions of the cerebral ganglia such as the pars intercerebralis.

The terminal ganglion (TermG) contains neuropeptides that are potentially involved in contractions of the hindgut and share several neuropeptides with the FG, such as AstC, CT-DH, MIP, MS, NVP, proctolin, sNPF, SIFa, and TKRP (Figure 1E,F). We also detected AT, IDL-containing (IDL), Pea-NPLP, and NPLP1 in the terminal ganglion of the two tenebrionids.

The RCC is the main neuroendocrine organ of insects and is connected to the cerebral ganglia, hypocerebral ganglion, FG, and gnathal ganglion. This organ consists of a pair of corpora cardiaca (CC), which are fused posteriorly to a pair of corpora allata. The CC store and release hormones and, in the glandular part, produce the adipokinetic hormone (AKH). Direct tissue profiling of the CC detected mostly AKH, MS, MIP, Pea-NPLP, pyrokinin (PK), and lower intensity signals matching with AstC, CT-DH, and NVP (Figure 2A,B). We also detected signals belonging to the CAPA precursor, such as CAPA-tPK, CAPA-PK, and the N-terminal truncated forms of PVK-1 and -2. In addition, we detected peptides below 10,000 Da matching with PPs of protein hormones such as ILP, ITP, and PTTH.

The thoracic and abdominal neurohemal organs of tenebrionids are different from those of other insects and other Coleoptera such as *Carabus*.^18,46^ The posterior lateral cells (PLCs) of the thoracic ganglia and the Va cells of the abdominal ganglia have axons projecting into segmental nerves (SNs), reaching paired neurohemal areas where neuropeptides are normally stored and released.^46^ The products of the PLCs entering the SN are mostly FMRFamide-related peptides (FMRF) (Figure 2C,D), whereas the Va cells produce CAPA peptides (Figure 2E,F). In the thoracic segmental neurohemal area of *T. molitor*, we also detected an abundant signal of MS and AstCC (Figure 2C).

### Neuropeptide Precursor Processing Based on MALDI-TOF Direct Tissue Profiling

Below, we report only the main findings regarding processed neuropeptides in comparison with other Coleoptera species.

#### Adipokinetic Hormone

Mature AKH neuropeptide was detected in the corpus cardiacum in the typical N-terminal blocked form only with Na^+^ and K^+^ adducts. Several intermediates of AKH have been described in other insect species containing the amidation signal (GK)^76−78^ and the dibasic cleavage motif (GKR),^76,77^ none of which we could confirm here.

#### Allatostatin C and CC

In holometabolan insects, allatostatin C and CC^18,24^ are the only two confirmed paralogs. AstC was detected with both the N-terminal blocked and not blocked forms, mostly in neuropil regions (see Figure 1). Previous immunochemical examination of the *T. molitor* CNS also showed the presence of AstC in the brain, the corpora allata, and the ventral nerve cord.^79^

#### Allatotropin

In both species, the consensus C-terminus of AT is typical for polyphagous Coleoptera (TARGYamide), i.e., contains a Tyr residue. Ion signals matching those of AT were detected in several preparations in the CNS of the two tenebrionids, especially in abdominal transverse nerves. In MALDI-TOF spectra, the ion signal intensities of the mature AT were often of relatively low abundance.

#### Calcitonin-Like Diuretic Hormone

The only precursor that was confirmed by mass spectrometry across several CNS tissues was the typical precursor containing CT-DH. Veenstra^8^ reported two additional splicing variants for CT-DH (=DH31) in *T. molitor* that were not detected in our mass spectrometry analyses.

#### CAPA

The *capa* gene encodes for periviscerokinins (PVKs), PKs and tryptopyrokinins (tPK) and is typically expressed by neurosecretory cells of the abdominal ganglia.^80^ Differential processing of this gene in the giant mealworm beetle *Z. atratus* was recently reported. The *capa* gene is expressed both in the neurosecretory cells of the gnathal ganglion, processing mostly CAPA-tPK and CAPA-PK, and in Va cells of the abdominal ganglia, in which mostly PVKs are processed.^46^ The differential processing of *capa* has also been reported in other insects, such as *T. castaneum*,^81^ the tobacco hawk moth *Manduca sexta*,^82^ the kissing bug *Rhodnius prolixus*,^83^ and ground beetles of the genus *Carabus*.^18^ In *T. molitor*, we could confirm the differential processing of the CAPA precursor in the gnathal ganglion with ion signals matching with mature CAPA-tPK and the CAPA-PK in CC preparations (Figure 2A). The latter peptide, which represents the third putative receptor ligand encoded by the CAPA precursor, was less abundant in *T. molitor* CC than in *Z. atratus* across several preparations (Figure 2A,B). The two PVKs of the CAPA precursors were both processed as predicted in the abdominal SNs, including additional N-terminally extended forms of PVK-1 (Figure 2E,F). The differential processing of the CAPA precursor is likely associated with mechanisms of inactivation of CAPA-tPK in abdominal Va cells^46^ and PVKs in CAPA cells of the gnathal ganglion.^18^ The C-terminally extended CAPA-tPK (ext. CAPA-tPK) was the most abundant form in the abdominal SNs. As far as the C-terminal consensus sequence of this peptide is not fully processed, it is likely inactive in abdominal ganglia (Figure 2E,F). Similarly, PVKs were detected in CC preparations only with a truncated C-terminus and thus likely inactive (Figure 2A,B). The inactivation of PVKs in CAPA cells of the gnathal ganglion by the truncation of the C-terminus had already been reported in *Carabus*(18) and in *D. melanogaster*,^84^ suggesting that this is a common feature, at least in holometabolan insects.

#### CCHamide

The CCHa-1 has two peptides resulting from a differential cleavage of the precursor signal peptide. The two peptides have a similar mass to peptides of the NPLP1 precursor and were sequence-confirmed only in preparations of the terminal ganglia nerves.

#### Corticotropin-Releasing Factor-Like Diuretic Hormone

The two transcripts^8^ of this gene were confirmed, and the shorter PP (CRF-DH-47-PP) of this gene was detected in several preparations across the CNS, especially in AL (Figure 1C,D) and FG (Figure 1A,B).

#### FMRFamide-Related Peptides

The precursor sequences for both species are currently complete, including the signal peptide of *T. molitor*, and mature FMRF neuropeptides were mostly detected in the neurohemal areas of the thoracic ganglia. FMRF-1 and -4 in *Z. atratus* were the only ones detected with an N-terminal extended form, which is the result of less efficient cleavage at the Arg residues. Among the six mature neuropeptides, the relative signal intensities were similar across different preparations, suggesting that potentially all six FMRF paracopies are equally expressed.

#### Myoinhibitory Peptide

Mature products of the *mip* gene were detected across the whole CNS, except ventral neurohemal areas, with MIP-5 being the peptide with the highest signal intensity in the MALDI-TOF spectra of both species. Immunohistochemical examination using antibodies against Drome-MIP also detects MIP immunoreactive neurons in the brain, along the ventral nerve cord and in the RCC of *T. molitor*.^79^

#### Myosuppressin

The sequence of the mature myosuppressin neuropeptide is one of the most conserved across insects, while the rest of the precursors (signal peptide and precursor peptide) are more variable, as shown in Polyneoptera insects.^85^ In tenebrionids, there is an additional cleavage site (Arg–Arg) along the precursor, suggesting the presence of at least two PPs. Moreover, in the *Z. atratus* precursor, there is an insertion of eight amino acids in the second PP, absent in *T. molitor* and *T. castaneum*. Myosuppressin was mostly detected in its pyroglutamate-blocked N-terminus form in both species across different tissues (Figure 1) in the MALDI-TOF spectra. We also sequenced a pyroglutamate-blocked form truncated at the C-terminus (MS^1−9^[pQ]), with similar mass match to MIP-2, and an N-terminal truncated form without the pyroglutamate-blocked Gln (MS^2−10^), both of which are from TermG preparations of *T. molitor* (Table S1). The second PP, where the insertion of eight amino acids is located, was confirmed only in *Z. atratus* but surprisingly was cleaved at a single Arg residue (Table S2) and not at the Arg–Arg site, producing a shorter than expected PP (MS-PP2^4−18^), abundant in preparations of the CC and FG (Figure 1; Table S2). The Arg–Arg cleavage site of tenebrionids is possibly not processed due to the presence of an aliphatic amino acid (Val) in the *plus* one position.^86^ Notwithstanding that we did not detect the homologous second PP in *T. molitor*, we could confirm a truncated PP cleaved at the single Arg residue (MS-PP^1−42^), suggesting that the same cleavage site was used in *T. molitor* (Table S1).

#### Pigment Dispersing Factor

The *pdf* gene was only recently described and confirmed by mass spectrometry in Coleoptera.^8,18^ In *Carabus*, the PDF was detected both in cerebral ganglia pars lateralis closer to the optic lobes and in the RCC.^18^ In tenebrionids, we could confirm the PDF only in cerebral ganglia preparations, but we could not detect any ion signal in the RCC.

#### Proctolin

In *T. molitor*, two genes encode for a single, not amidated peptide well conserved across insects, which is typically abundant in the SNS. In *T. molitor* preparations of both FG and TermG, we could confirm the proctolin peptide and the complete PP of proctolin 1, including a truncated form cleaved at unexpected residue (proctolin 1 – PP2^28−42^) (Figure 1F, Table S1). The proctolin 2 allele 1 (PP2) was only confirmed by MS^1^, whereas using enzymatic digestion, we could also detect few peptides matching with fragments of the PP of both proctolin 2 alleles 1 and 2 (Supporting Information S1).

#### *Periplaneta* Neuropeptide-Like Precursor (=PaOGS36577)

This neuropeptide-like precursor was detected in FG, AL, RCC, and TermG preparations of both species (Figures 1 and 2), similar to those already reported in *P. americana*(68) and in *C. nodus*.^69^

#### Pyrokinin

The *pk/pban* gene of *Z. atratus* now includes the nearly complete signal peptide and tPK peptide (Table S2).^18,22,46^ tPK typically have the consensus sequence of MWFXPRLamide, with the characteristic constant presence of Trp at position 6 from the C-terminus followed by the pentapeptide FXPRL motif typical of PKs.^70^ In *Z. atratus*, the C-terminus consensus sequence of tPK is derived compared to other beetles including tenebrionids,^8^ with the Phe^5^ replaced by a Pro and the Arg^2^ replaced by a Lys. Using mass spectrometry, we could confirm the atypical tPK of *Z. atratus* and all processed *T. molitor* neuropeptides. Interestingly, the presence of both tPK was confirmed in *Z. atratus* CC, one encoded by the *capa* gene, having a typical C-terminus (...MWFGPRL-NH_2_), and one with the atypical C-terminus encoded by the *pk/pban* gene (...VWPSPKL-NH_2_). As these two tPK have relatively different C-terminus consensus sequences, it would be interesting to test if they also differ in ligand–receptor interactions.

#### Short Neuropeptide F

This was one of the most abundant peptides detected in our preparations, especially in the FG and TermG. Similarly to what was reported in *Carabus*,^18^ the internal Arg residue, which is used as an internal cleavage site in several insects,^19,76,87^ is not efficient in cleaving the mature peptide.

#### Protein Hormones

**Figure 3:**
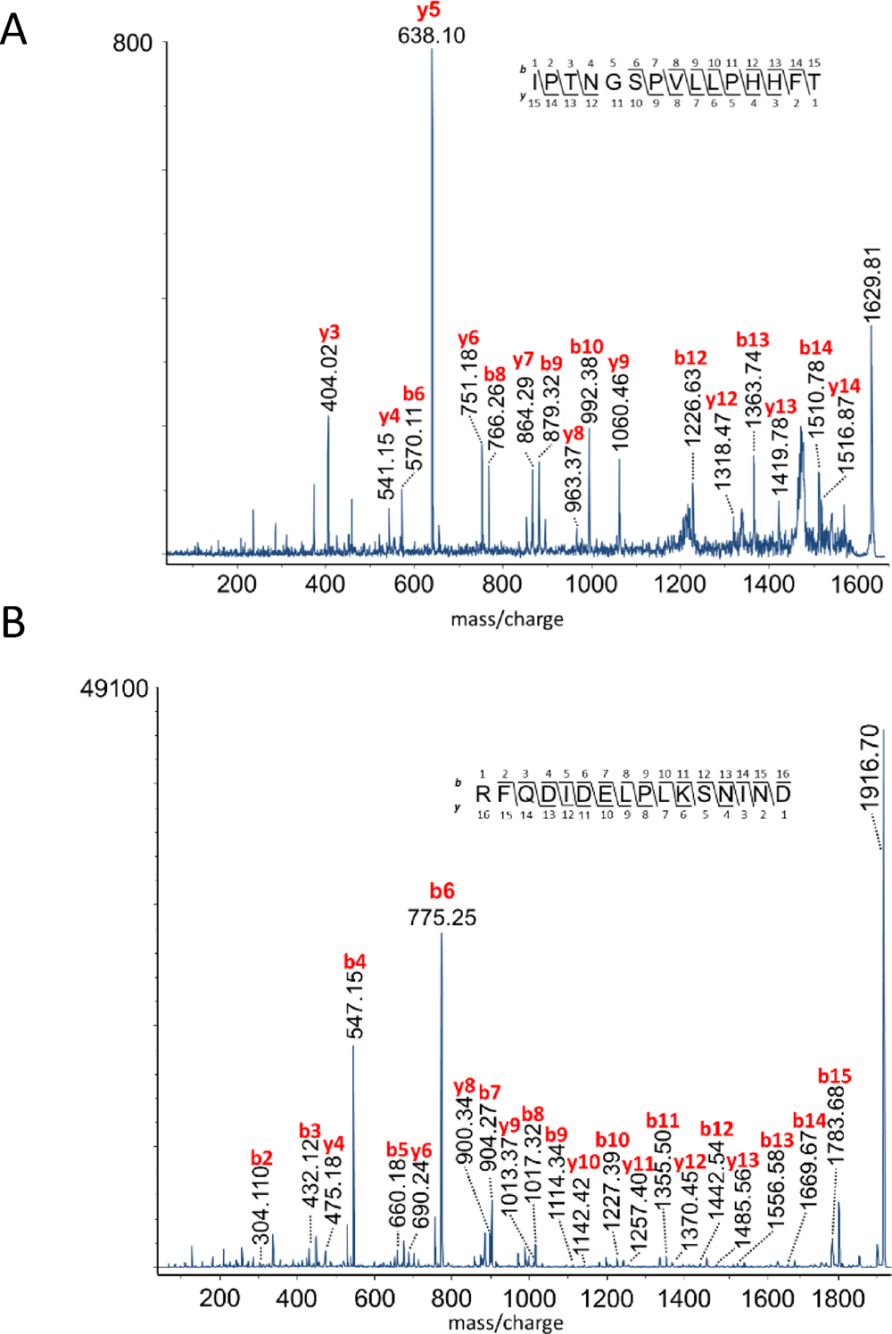
MALDI-TOF MS^2^ spectrum of (A) *T. molitor* ITP-PP1 and (B) *Z. atratus* PTTH-PP2, both from preparations of the RCC. Ion signals of *b*- and *y*-type fragment ions are labeled.

Large proteins are rarely detected in MALDI-TOF direct tissue profiling because they are outside of the mass range used to analyze neuropeptides and neuropeptide-like hormones. In our MALDI-TOF spectra, we detected few short PPs, which suggest the presence of these large proteins mostly in preparations of the RCC, such as ILP, ITP, and PTTH (Tables S1 and S2; Figure 3). ILP peptides had already been reported in other insects,^18,88^ whereas we detected an ITP precursor peptide (Figure 2B) matching with a predicted alternative cleavage site of the signal peptide; in the case of PTTH, we detected a peptide (Figure 2B) cleaved at two Lys–Lys cleavage sites.

## Conclusions

Using transcriptomic and mass spectrometry analyses of two tenebrionid species, we were able to obtain a comprehensive list of neuropeptide, neuropeptide-like, and protein hormones and their distributions across the CNS of *T. molitor* and *Z. atratus*. The present study is one of the most detailed neuropeptidomic surveys within Tenebrionidae and Polyphaga beetles. The neuropeptidomes of the two studied species are similar, and the information obtained about the distribution of neuropeptides in the CNS should be similar in closely related tenebrionid species commonly used in genetics, immunology, and developmental biology, such as *T. castaneum*. This study also provides the basis for functional studies of neuropeptides in these two model organisms for physiology. For instance, FMRF-6 is identical between these two species and has already been tested in both species showing myostimulatory activity of visceral muscles (i.e., heart, hindgut, and oviduct) in a dose-dependent manner.^89^ Similarly, myosuppressin was shown to modulate muscle contractility in different insects, including beetles,^90−92^ and to regulate digestive system function.^93^

During recent years, neuropeptide analogues of insect kinin, TKRP, PKs, and sulfakinins have been successfully tested as greener insecticides.^30−35,94^ Based on the current list of mass spectrometrically confirmed neuropeptides, it would be possible to design and test additional neuropeptide analogues targeting key physiological and behavioral mechanisms of selected species in order to control pests in cultivated fields and grain storage without affecting nontargeted beneficial species. At the same time, since *T. molitor* larvae have been accepted as novel food, increased knowledge on their genomes, genes, and neuropeptidomes could prove useful to optimize programs for the production of this species, as well as other tenebrionids, at the industrial scale.^47,48^

## Supporting Information Available

Table S1. Mature neuropeptides and additional precursor peptides (PPs) of *T. molitor* identified by mass spectrometry. Table S2. Mature neuropeptides and additional precursor peptides (PPs) of *Z. atratus* identified by mass spectrometry. Supporting information S1: List of neuropeptide precursors, neuropeptide-like precursors and protein hormone precursors from *T. molitor* and *Z. atratus*. Figure S1: Splice forms in *T. molitor*. Figure S2: Orcokinin-like precursor alignments of *T. castaneum*, *T. molitor* and *Z. atratus*. Figure S3: Proctolin 2 alleles alignment of *T. molitor*. Figure S4: Splice forms in *Z. atratus*(95) (PDF)
