# Supplementary material for: Neuropeptidomes of Tenebrio molitor L. and Zophobas atratus Fab. (Coleoptera, Polyphaga: Tenebrionidae)
Source: J Proteome Res. 2022 Sep 15;21(10):2247–60. doi: 10.1021/acs.jproteome.1c00694 (PMC9552230; doi:10.1021/acs.jproteome.1c00694)

Supporting information:

Neuropeptidomes of *Tenebrio molitor* L. and *Zophobas atratus* Fab. (Coleoptera, Polyphaga:Tenebrionidae)

Paweł Marciniak[1*], Joanna Pacholska-Bogalska[1], Lapo Ragionieri[2*]

[1]Department of Animal Physiology and Developmental Biology, Institute of Experimental Biology, Faculty of Biology, Adam Mickiewicz University, Poznań, Poland

[2]University of Cologne, Department of Biology, Institute for Zoology, Zülpicher Str. 47b, 50674 Cologne, Germany

Corresponding authors:

[*]Paweł Marciniak pmarcin@amu.edu.pl

[*]Lapo Ragionieri lapo.ragionieri@uni-koeln.de

Table of contents:

Table S1. Mature neuropeptides and additional precursor peptides (PPs) of *T. molitor* identified by mass spectrometry……S2-S5

Table S2. Mature neuropeptides and additional precursor peptides (PPs) of *Z. atratus* identified by mass spectrometry……S6-S9

Supporting information S1: List of neuropeptide precursors, neuropeptide-like precursors and protein hormone precursors from *T. molitor* and *Z. atratus*……S10-S20

Figure S1: Splice forms in *Tenebrio molitor*. ……S21-S23

Figure S2: Orcokinin-like precursor alignments of *T. castaneum*, *T. molitor* and *Z. atratus*. ……S24

Figure S3: Proctolin 2 alleles alignment of *T. molitor*. ……S25

Figure S4: Splice forms in *Zophobas atratus*. ……S25

Table S1. Mature neuropeptides and additional precursor peptides (PPs) of *T. molitor* identified by mass spectrometry (direct tissue profiling with MALDI-ToF MS and with Q-Exactive Orbitrap MS). Distinct peptides from different transcripts are marked with subscripts (e.g. CAPA$_a$, CAPA$_b$). Cysteines which form disulfide bridges underlined.

Designation	Peptide sequence	m/z [M+H]$^+$	MALDI TOF MS^2	Orbitrap MS^2
**Adipokinetic hormone (AKH)**				
AKH-1[pQ]*	QLNFSPNW-NH$_2$	1009.45	MS^2	MS^2
AKH-1[pQ]¥	QLNFSPNW-NH$_2$	1025.47	MS^2	-
AKH-2-PP	VSSTTGGGESDNCKESMDVIMLIYKLIQNEAQKLVECEK FSN-OH	4636.18	MS^1	MS^2
**Adipokinetic hormone/Corazonin related Peptide (ACP)**				
ACP [pQ]	QVTFSRDWNP-NH2	1231.59	MS^1	MS^2
**Allatostatin-C (AstC)**				
ASTC	QSRYRQCYFNPISCF-OH	1909.84	MS^2	MS^2
ASTC[pQ]	QSRYRQCYFNPISCF-OH	1892.82	MS^2	MS^2
ASTC-PP	RPNHFGDPNQVVAEGDANNLLDSGLKPWQLEMLAQRLSE ISQTGGDYGWDKSIRSPES-OH	6423.11	MS^1	MS^2
**Allatostatin-CC (AstCC)**				
ASTCC	GHGSMSGQQKGRVYWRCYFNAVTCF-OH	2880.29	MS^2	MS^2
ASTCC-PP1	SAASERNSDDYPDYQLGVKYDEYPMIVPK-OH	3350.55	MS^2	MS^2
ASTCC-PP1$^{1-28}$	SAASERNSDDYPDYQLGVKYDEYPMIVP-OH	3222.45	MS^2	MS^2
ASTCC-PP1	TALLVDRLMVALQQAIEEEEAANRVDGPPLTDSFSLSPE EVRKMDLQ-OH	5224.65	MS^1	MS^2
**Allatotropin (AT)**				
AT	GIEHFKYHNMDLGTARGY-NH2	2108.01	MS^1	MS^2
AT-PP2	AVDMHNVNNFLLEWIALETRMRDLGIPRNLLRDQETIPE -OH	4632.38	-	MS^2
**Anti diuretic factor (ADF)**				
ADF-1	YDDGSYKGE-OH	1033.41	-	MS^2
**Calcitonin-like diuretic hormone (CT-DH31)**				
CT-DH	GLDLGLGRGFSGSQAAKHLMGLAAANFAGGP-NH$_2$	2940.52	MS^2	MS^2
CT-DH-PP	APHSSRYYPGYYSPLSMEGQNPEYLLQTIARLRQALIAD DDLENS-OH	5142.49	MS^1	MS^2
**CAPA**				
ext. PVK-1	ESKEPKRSKLSSVYALTPSLRV-NH$_2$	2474.40	MS^2	-
ext. PVK-1$^{1-20}$	ESKEPKRSKLSSVYALTPSL-OH	2220.22	MS^1	-
ext. PVK-1$^{3-22}$	KEPKRSKLSSVYALTPSLRV-NH$_2$	2258.33	MS^2	-
ext. PVK-1$^{3-20}$	KEPKRSKLSSVYALTPSL-OH	2004.15	MS^2	MS^2
PVK-1	SKLSSVYALTPSLRV-NH$_2$	1619.94	MS^2	MS^2
PVK-1$^{1-13}$	SKLSSVYALTPSL-OH	1365.76	MS^1	MS^2
ext. PVK-2-G	RIGKMVSFPRIG-OH	1360.79	MS^2	-
PVK-2	RIGKMVSFPRI-NH$_2$	1302.78	MS^2	-
PVK-2$^{1-10}$	RIGKMVSFPR-OH	1190.68	MS^2	MS^2
ext. PVK2$_b$	SDDSWDPNTDVKRRIGKMVSFPR-OH	2706.34	MS^1	-
CAPA-tPK	GDSNWVADENNYGAKRPGANSGMWFGPRL-NH$_2$	3165.47	MS^2	MS^2
ext. CAPA-tPK	GDSNWVADENNYGAKRPGANSGMWFGPRLGRLQ-OH	3620.72	MS^1	MS^2
CAPA-PK	QVHYTPRL-NH$_2$	1012.57	-	MS^2
CAPA-PK[pQ]	QVHYTPRL-NH$_2$	995.54	-	MS^2
CAPA$_a$-PP1	SDDSWDPNTGRQPSGAAPAHFARLADV-OH	2838.31	MS^1	MS^2
CAPA$_b$-PP1	SDDSWDPNTDV-OH	1250.48	-	MS^2
CAPA-PP4	ESDEVYDELDADVDVLA-OH	1896.82	MS^2	-
**CCHamide 1 (CCHa1)**				
CCHa1	SCLSYGHACWGAH-NH$_2$	1388.56	MS^2	MS^2
CCHa1	GSCLSYGHACWGAH-NH$_2$	1445.58	MS^2	MS^2
**CCHamide 2 (CCHa2)**				

CCHa2	KRGCATFGHSCYGGM-NH$_2$	1571.66	MS^1	MS^2
CCHa2	GCATFGHSCYGGM-NH$_2$	1287.47	-	MS^2
**Corticotropin-releasing factor-like diuretic hormone (CRF-DH)**				
CRF-DH-37	SPTISITAPIDVLRKTWEQERARKQMVKNREFLNSLN-OH	4369.36	MS^2	MS^2
CRF-DH-37-PP	EDHNYGRLLEPIDVAADQETVSYLLPKLTAKYRPNNEWS SVTDPRFYVLTEMESNDIDNQMPSERSIQ-OH	7927.81	MS^1	-
CRF-DH-47	AGALGESGASLSIVNSLDVLRNRLLLEIARKKAKEGANR NRQILLSL-NH$_2$	5026.91	MS^1	-
CRF-DH-47-PP	AFLQSRASGTYDNNV-OH	1642.78	MS^2	MS^2
**Crustacean cardioactive peptide (CCAP)**				
CCAP-PP	LFLPKSLGQNLAARERVVEPK-OH	2365.37	MS^1	MS^2
**Elevenin**				
Elevenin	SQFSLTSYCRKHILSPPCRGHQ-OH	2543.24	MS^2	MS^2
Elevenin-PP1	SAIKGPDNE-OH	930.45	-	MS^2
**FMRFamide-related peptides (FMRFa)**				
FMRF-1	NNNNFLRF-NH$_2$	1037.53	MS^2	MS^2
FMRF-2	SGKTEKNDHFIRF-NH$_2$	1577.82	MS^2	MS^2
FMRF-3	SKQDFLRF-NH$_2$	1039.57	MS^2	MS^2
FMRF-4	DQHRVVRDRSGNYLRF-NH$_2$	2017.06	MS^2	MS^2
FMRF-5	GGSNFMRF-NH$_2$	914.43	MS^2	MS^2
FMRF-6	NSNFLRF-NH$_2$	896.47	MS^2	MS^2
FMRF-PP3	SVPNTEEKVRN-OH	1272.65	MS^1	MS^2
FMRF-PP5	NNEMTATSDPEKVQQLQESPLVQLLSELLEHIKKGQDKN RIV-OH	4800.52	-	MS^2
**HanSolin**				
HanSolin$_{ext}$	SNPNTLPFNQIRDRQHGQPLRW-NH$_2$	2673.38	-	MS^2
**IDL-containing (IDL)**				
IDL	IDLSRLYGHVNS-OH	1373.72	MS^1	MS^2
**Inotocin**				
Inotocin	CLITNCPRG-NH$_2$	973.47	MS^1	-
**Insect parathyroid hormone (iPTH)**				
iPTH-PP	SQSRFLDVLFNHSEEDKGDPVDFNDYESLIQRFRNLE-OH	4459.12	-	MS^2
**Myoinhibitory peptides (MIP)**				
MIP-1	DWNKDLHIW-NH$_2$	1225.61	MS^2	MS^2
MIP-2	GWNNLHEGW-NH$_2$	1111.51	MS^2	MS^2
MIP-3	AWQNLHSGW-NH$_2$	1097.53	MS^2	MS^2
MIP-4	NWGQFHGGW-NH$_2$	1087.49	MS^2	MS^2
MIP-5	SKWDNFRGSW-NH$_2$	1281.61	MS^2	MS^2
MIP-6	EPAWSNLKGIW-NH$_2$	1299.68	MS^2	MS^2
MIP-6 [pE]	EPAWSNLKGIW-NH$_2$	1282.66	-	MS^2
MIP-5-6	SKWDNFRGSWGKREPAWSNLKGIW-NH$_2$	2904.48	-	MS^2
MIP-PP1	L**S**DETPMKSTNDNPQMDDEMS-OH	2384.95	MS^1	MS^2
MIP-PP2	SAPSWGDQPAME-OH	1275.53	-	MS^2
MIP-PP3	FAPEDEYAIRQLAAMLEPQYDEYNPEGDLDVNDDE-OH	4073.79	-	MS^2
MIP-PP4	SVQDQIAQ-OH	888.44	-	MS^2
**Myosuppressin (MS)**				
MS[pQ]	QDVDHVFLRF-NH$_2$	1257.66	MS^2	MS^2
MS$^{1-9}$[pQ]	QDVDHVFLR-OH	1111.55	MS^2	MS^2
MS	QDVDHVFLRF-NH$_2$	1274.66	MS^2	MS^2
MS$^{2-10}$	DVDHVFLRF-NH$_2$	1146.61	MS^2	MS^2
MS-PP	TAISCPPNPLEASPYVRHLCYAIEQAISENAITDDQYRR VEERNVNGNA-OH	5485.63	MS^1	MS^2
MS-PP $^{1-42}$	TAISCPPNPLEASPYVRHLCYAIEQAISENAITDDQYRR VEE-OH	4760.27	MS^1	MS^2
**Natalisin (Nat)**				
NAT1	SGQDEFGPFWANR-NH$_2$	1509.69	-	MS^2
NAT2[pQ]	QNVLQNLADLPIFIEQDRK-NH$_2$	2236.21	-	MS^2

**Neuropeptide F1 (NPF1)**				
NPF1a	APSPRNDDMFKELLRLDQMYSSIARPRF-NH_2	3352.70	MS^1	-
**Neuropeptide F2 (NPF2)**				
NPF2	RECNRPKNSFENIKHVHEYLECMKSQVSTRY-NH_2	3822.83	MS^1	-
**Orcokinin-like (OK-like)**				
OK-like-PP1	APNLARLESNYNPYGEIQSVM-NH_2	2365.16	-	MS^2
OK-like-PP3	GPLNGLIPGGAF-NH_2	1111.62	-	MS^2
**Pigment dispersing factor (PDF)**				
PDF	NSEVSNAIMGSEETQKMYRD-NH_2	2288.03	MS^2	MS^2
PDF-PP	YPSPGDDYRYLDRDYASPGAHQLASWIASQLRPKEYAPA PEVPILPYRLPLQ-NH_2	5940.03	MS^1	MS^2
**Proctolin**				
Proctolin	RYLPT-OH	649.37	MS^2	-
Proctolin1-PP1	LETRHTVEA-OH	1055.54	-	MS^2
Proctolin1-$PP2^{28-42}$	DAPQRWHPENKLFY-OH	1800.88	MS^2	-
Proctolin1-PP2	SNGDRVDKLRELLKDLLQSEIEKEEYQADAPQRWHPENK LFY-OH	5100.58	MS^2	MS^2
Proctolin2-PP2 allele 1	SNGDRVDKLRELLRDLLEKELDDGYQGYVFSKWHPENKL SYNK-OH	5167.62	MS^1	-
**Pyrokinins (PK)**				
tPK	RERNDDKKQSYMWFGPRL-NH_2	2325.17	MS^2	MS^2
tPK^{4-18}	NDDKKQSYMWFGPRL-NH_2	1883.92	MS^2	MS^2
tPK^{8-18}	QSYMWFGPRL-NH_2	1283.64	-	MS^2
tPK [pQ] $^{8-18}$	QSYMWFGPRL-NH_2	1266.61	MS^1	-
PK-1	HVVNFTPRL-NH_2	1081.63	MS^2	MS^2
PK-1_ext	NSNIDPYRNREREQLATLLDVIQDSPWAIVAVNGKRHVV NFTPRL-NH_2	5213.78	MS^1	MS^2
PK-2	SPPFAPRL-NH_2	883.51	MS^2	MS^2
PK-3	HLSPFSPRL-NH_2	1052.60	MS^2	MS^2
PK-PP1	VPHYGSHQVSV-OH	1209.60	MS^1	MS^2
PK-PP2	NSNIDPYRNREREQLATLLDVIQDSPWAIVAVN-NH_2	3808.97	MS^2	MS^2
PK-PP3	ESGEEFVSSAAEDRWLQDPEMSGEMLSQ-OH	3144.34	MS^2	MS^2
PK-PP4	ENDKNLF-OH	879.42	MS^1	MS^2
**RYamide (RYa)**				
RYa-1	VQNLSTFKTMMRY-NH_2	1617.82	MS^1	-
RYa-2	AGPNPNEKESKVNIHPRADAFFLGPRY-NH_2	3023.56	MS^1	MS^2
**Short neuropeptide F (sNPF)**				
sNPF	SSRSPSLRLRF-NH_2	1304.75	MS^2	MS^2
$sNPF^{4-11}$	SPSLRLRF-NH_2	974.59	MS^1	-
sNPF-PP1	APSYADYDNNIRDLWEMLLQKDALEDKFGGHQMV-OH	3982.87	-	MS^2
sNPF-PP2	SDSSMSPEAAFMMAQAVDHDN-OH	2240.89	MS^1	MS^2
**SIFamide (SIFa)**				
SIFa	TYRKPPFNGSIF-NH_2	1425.76	MS^2	MS^2
Ext. SIFa	EATYRKPPFNGSIF-NH_2	1625.84	-	MS^2
**Sulfakinin (SK)**				
SK-1[pQ](SO_3)	pQTSDDY(SO_3)GHLRF-NH_2	1400.56	-	MS^2
SK-1	QTSDDYGHLRF-NH_2	1337.62	MS^1	-
SK-2 (SO_3)	GEEPFDDY(SO_3)GHMRF-NH_2	1678.63	-	MS^2
SK-2	GEEPFDDYGHMRF-NH_2	1598.67	MS^2	MS^2
**Tachykinin-related peptides (TKRP)**				
TKRP-1	APSGFTGVR-NH_2	890.48	MS^1	MS^2
TKRP-2(x3)	APSGFMGMR-NH_2	952.45	MS^2	MS^2
TKRP-5	APMGFVGMR-NH_2	964.47	MS^1	MS^2
TKRP-6	APSGFFGMR-NH_2	968.48	MS^1	MS^2
TKRP-7	MPRQSGFFGMR-NH_2	1312.64	MS^2	MS^2
TKRP-8	YPYEFRGKFVGVR-NH_2	1616.87	MS^2	MS^2
TKRP-PP1	SIPDSAYSTGNAESDATSELKADVVSDVGAVN-OH	3169.47	MS^1	MS^2
TKRP-PP2	PYPVWEGTYPDEVY-OH	1714.76	MS^2	MS^2
TKRP-PP3	DMEFTNYGDEYD-OH	1498.53	-	MS^2

TKRP-PP4	EYDSLSNQYDGYFE-OH	1729.69	-	MS^2
**NEUROPEPTIDE-LIKE**				
**Agatoxin like (ALP)**				
ALP$_b$	ACVRRGGNCDHRPNDCCYNSSCRCNLWGSNCRCQRMGLF QKW-NH$_2$	4888.01	MS^1	-
ALP-PP	GPYLEDDEGLPSDDDYTENAIDRLLQSAQ-OH	3239.45	MS^2	MS^2
**Neuropeptide-like precursor 1 (NPLP1)**				
	SLSSLAQWDNLPD-OH	1445.70	-	MS^2
	NLEALARAGYVRTLPSQDDEDPNY-OH	2707.30	-	MS^2
	SLATLAKNGQLPTYQNNDS-OH	2035.00	-	MS^2
	GIESLARNGELTT-OH	1360.71	-	-
	GIESLARNGELTTRREIQELLDELYNKRNVGSLARNFNF PTY-NH$_2$	4896.55	-	MS^2
	NVGSLARNFNFPTY-NH$_2$	1598.81	MS^2	MS^2
	FLGSLVRNGDSQYS-NH$_2$	1541.77	MS^1	MS^2
	NIASLAREGGRFV-NH$_2$	1388.78	MS^2	MS^2
	NVAAMLRQDNYLNGQKSNEKVEGPELDNE-OH	3275.55	MS^1	MS^2
	NLASIKAQYSGKF-OH	1426.77	-	MS^2
	FLGSVAKTGWFRPTSRYRSPE-OH	2442.26	MS^1	MS^2
	HIGALARLGWLPTLRNV-OH	1887.10	MS^1	MS^2
**NVP-like peptide (NVP)**				
	IPASLIEEIKASELRNNKV-OH	2124.20	MS^2	MS^2
	ASLIEEIKASELRNNKV-OH	1914.07	-	MS^2
	IEEIKASELRNNKV-OH	1642.91	-	MS^2
	IPASLIEEIKASELRNN-OH	1897.04	MS^2	MS^2
	AHPPMNNVEERSRDVPYYSKPTAI-OH	2771.36	MS^2	MS^2
	DARKIRPDNRI-OH	1353.77	MS^2	MS^2
	GRWGGFADN-OH	979.44	MS^2	MS^2
	GRWGGFA-OH	750.37	MS^1	MS^2
	NDPTRELRYLNGPNKNDYYTLSQLLSNQREPNVPLYHRL VL-OH	4913.54	MS^2	MS^2
**Periplaneta neuropeptide-like precursor (Pea-NPLP)**				
	AYYPRFGLDSVGL-OH	1457.74	MS^2	MS^2
	YDSRQKTYNSV-OH	1360.65	MS^1	MS^2
	NKYYDGDTLSDPPYLYNYNSRDDFYNKYEDEDDEKDVDS SYYDWARANKHY-OH	6334.69	MS^1	MS^2
	SSSFEPPRDSEITHEH-OH	1854.83	MS^1	MS^2
	SEDELDSEWLMERYHKAVGLTS-OH	2595.20	MS^2	MS^2
	NAEYPLQSFKNHDQPS-OH	1874.87	MS^1	MS^2
**PROTEIN HORMONES**				
**Glycoprotein hormone beta 5 (GPB)**				
GPB-PP	SHPASLGFRIN-OH	1198.63	MS^2	MS^2
**Insulin like peptide ILP (ILP)**				
ILP-1-PP1	SPHKLHSIN-OH	1032.56	MS^2	MS^2
ILP-1 (B-chain)	AEFFCGSKLSEALYMVCKGSYNSPT-OH	2730.22	-	MS^2
ILP-3-PP1	TPHMASFMN-OH	1035.44	-	MS^2
ILP-4$_1$ PP2	SSLGWNEWDGEKLQKEEQVGAID-OH	2618.24	MS^1	MS^2
ILP-4$_2$ PP2	SSLGWNEWDGEKLEKEEQVGAID-OH	2619.22	-	MS^2
**Ion transport peptide**				
ITP$_{a-b}$-PP	IPTNGSPVLLPHHFT-OH	1629.87	MS^2	MS^2
**ITG-like (ITG)**				
ITG-PP	LTGLASFKRPMH-OH	1357.74	MS^1	MS^2
ITG-PP$^{1-11}$	LTGLASFKRPM-OH	1220.68	MS^2	MS^2

*[M+Na]

¥[M+K]+.

Table S2. Mature neuropeptides and additional precursor peptides (PPs) of *Z. atratus* identified by mass spectrometry (direct tissue profiling with MALDI-TOF MS and with Q-Exactive Orbitrap MS). Cysteines which form disulfide bridges underlined.

Designation	Peptide sequence	m/z [M+H]+	MALDI TOF MS^1/MS^2	Orbitrap MS^2
**Adipokinetic hormone (AKH)**				
AKH[pQ]*	QLNFSPNW-NH_2	1009.45	MS^2	–
AKH[pQ]¥	QLNFSPNW-NH_2	1025.46	MS^2	–
AKH-PP	ATSSAGGENDNCKESVDTIMLIYKIIQNEAQKLVECE KFSN-OH	4518.14	MS^1	MS^2
**Adipokinetic hormone/Corazonin related Peptide (ACP)**				
ACP [pQ]	QVTFSRDWNP-NH_2	1231.69	MS^2	MS^2
ACP-PP	GENPDFHNAMKTASAVCHLLINQVRQLATCDNRDEIE PGANNIFG-NH_2	4921.32	MS^1	–
**Allatostatin C (AstC)**				
AST C	QSRYRQCYFNPISCF-OH	1909.84	MS^2	MS^2
AST C [pQ]	QSRYRQCYFNPISCF-OH	1892.82	MS^2	–
AST C-PP	NHFGEGNQVVAEQDGNNLLDPGLKPWQLELLAQRLSE ISSQTGGDYAWDRSLRSPEA-OH	6306.09	MS^1	–
**Allatostatin-CC (AstCC)**				
ASTCC	GHGSMGGQQKGRVYWRCYFNAVTCF-OH	2850.28	MS^2	–
AstCC-PP-1	SAASERSDDYPDYQLGVKYDEYPMIVP-OH	3108.41	MS^1	–
AstCC-PP-2	RTALLVDRLMVALQQAIEEEEAANRVDGPPLTDGYSL SPEEVRKMDLQ-OH	5366.74	MS^1	–
**Allatotropin (AT)**				
AT	GIEHLKYHNMDLGTARGY-NH_2	2074.03	MS^2	MS^2
AT-PP1	RRDSKYPQVRTPQQRLT-OH	2129.17	MS^1	–
AT-PP2	AVDMHNVNNFLLEWIALETRMRNLGIPRNLVRDQDTI PE-OH	4603.37	MS^1	–
**Antid diuretic factor (ADF)**				
ADF-2	YDDGSYKPHVYGH-OH	1537.67	–	MS^2
**Calcitonin-like diuretic hormone (CT-DH)**				
CT-DH	GLDLGLGRGFSGSQAAKHLMGLAAANFAGGP-NH_2	2940.53	MS^2	MS^2
CT-DH-PP	APHSSRYYPGYYSPLSMEGQNPEYLLQTIARLRQALI ADDDLENS-OH	5142.49	MS^1	–
**CAPA**				
PVK-1	SKLSSDFGLTPFLRS-NH_2	1653.89	MS^2	MS^2
PVK-1$^{1-13}$	SKLSSDFGLTPFL-OH	1411.75	MS^1	MS^2
ext. PVK-1	KEPKRSKLSSDFGLTPFLRS-NH_2	2292.28	MS^2	–
ext. PVK-1$^{1-18}$	KEPKRSKLSSDFGLTPFL-OH	2050.13	MS^2	MS^2
PVK-2	KIGKMVSFPRI-NH_2	1274.78	MS^2	–
PVK-2$^{1-10}$	KIGKMVSFPR-OH	1162.67	MS^2	MS^2
ext. PVK-2$^{1-10}$	GSEHSWDANVDVKRKIGKMVSFPR-OH	2743.41	MS^2	–
CAPA-tPK	SESWGADENNYGAKRPGANSGMWFGPRL-NH_2	3053.41	MS^2	–
ext. tPK	SESWGADENNYGAKRPGANSGMWFGPRLGRVQ-OH	3494.64	MS^2	–
CAPA-PK[pQ]	QVHYTPRL-NH_2	995.57	MS^2	MS^2
CAPA-PP2	GSEHSWDANVDV-OH	1315.55	MS^2	MS^2
CAPA-tPK ext.	PGANSGMWFGPRLGRVQ-OH	1829.9	MS^2	–
CAPA-PP4	SDEYTPWTYIIVNGEGPVT-OH	2141.01	–	MS^2
CAPA-PP5	ESEEVYDDLDAEDVA-OH	1698.69	MS^1	–
**CCHamide1 (CCH1a)**				
CCHa-1$^{2-14}$	SCLSYGHACWGAH-NH_2	1388.56	MS^1	–
CCHa-1	GSCLSYGHACWGAH-NH_2	1445.58	MS^2	MS^2

**CCHamide 2 (CCHa2)**				
CCHa-2$^{3-15}$	GCATFGHSCYGGM-NH$_2$	1287.47	-	MS^2
CCHa-2	KRGCATFGHSCYGGM-NH$_2$	1571.66	-	MS^2
CCHa-2-PP	AQELHADNNI-OH	1124.53	-	MS^2
**Corticotropin-releasing factor-like diuretic hormone (DH37)**				
DH-37	SPTISIAAPIDVLRKTWEQERARKQMLKNREFLNSLH-OH	4376.38	MS^1	-
**Corticotropin-releasing factor-like diuretic hormone (DH47)**				
DH-47-PP	AFLQSRASGNYDNNV-OH	1655.78	MS^2	MS^2
**Crustacean Cardio-Active Peptide (CCAP)**				
CCAP	PFCNAFTGC-NH$_2$	956.37	MS^2	-
**FMRFamide related peptides (FMRF)**				
FMRF-1	NNNNFLRF-NH$_2$	1037.52	MS^2	MS^2
FMRF1 + PP1	YNEELYPLSDPDSSYLYPEDVPDETEFEIHRRNNNNFLRF-NH$_2$	4936.28	MS^1	MS^2
FMRF-2	SGKIDKNNDFFIRF-NH$_2$	1699.89	MS^2	-
FMRF-3	SKQDFLRF-NH$_2$	1039.56	MS^2	MS^2
FMRF-4$^{8-16}$	AKNEFHLRF-NH$_2$	1160.63	MS^2	MS^2
FMRF-4	DQERPVRAKNEFHLRF-NH$_2$	2041.08	MS^2	-
FMRF-5	AGSNFLRF-NH$_2$	910.48	MS^2	-
FMRF-6	NSNFLRF-NH$_2$	896.47	MS^2	MS^2
FMRF-PP1	YNEELYPLSDPDSSYLYPEDVPDETEFEIH-OH	3605.56	MS^2	-
FMRF-PP1$^{3-30}$	EELYPLSDPDSSYLYPEDVPDETEFEIH-OH	3328.46	MS^1	-
FMRF-PP2	SGRKYDSEYEDYNEDFARPT-OH	2442.05	MS^2	-
**IDL-containing (IDL)**				
IDL	IDLSRLYGHISS-OH	1360.72	-	MS^2
IDL$^{1-11}$	IDLSRLYGHIS-OH	1273.69	MS^2	MS^2
**Inotocin**				
Inotocin	CLITNCPRG-NH$_2$	973.46	MS^2	-
**Myoinhibitory peptides (MIP)**				
MIP-1	DWNKDLHIW-NH$_2$	1225.61	MS^2	MS^2
MIP-2	GWNNLHDGW-NH$_2$	1097.49	MS^2	MS^2
MIP-3	AWQNLHSGW-NH$_2$	1097.53	MS^2	MS^2
MIP-4	NWGQFHGGW-NH$_2$	1087.49	MS^2	MS^2
MIP-5	SNWGNFRGSW-NH$_2$	1209.55	MS^2	-
MIP-6	EPAWSNLKGIW-NH$_2$	1299.68	MS^2	-
ext. MIP-PP1	LSDETPMKSSNDNPQMDYDMS-OH	2404.96	MS^1	-
PP2$^{2-11}$	SVPSWADQAD-OH	1075.47	-	MS^2
PP4	SQDQIAQ-OH	789.37	-	MS^2
**Myosuppressin (MS)**				
MS[pQ]	QDVDHVFLRF-NH$_2$	1257.88	MS^2	-
MS	QDVDHVFLRF-NH$_2$	1274.66	MS^1	MS^2
MS-PP1	STTAYMISCPPNDLLEASPSLRHLCYIVEKAVVDNSISDEPSY-OH	4726.23	-	MS^2
ext. MS-PP1	STTAYMISCPPNDLLEASPSLRHLCYIVEKAVVDNSISDEPSYRRVVE-OH	5365.61	MS^1	MS^2
MS-PP1$^{5-43}$	YMISCPPNDLLEASPSLRHLCYIVEKAVVDNSISDEPSY-OH	4366.07	-	MS^2
ext. MS-PP1$^{5-48}$	YMISCPPNDLLEASPSLRHLCYIVEKAVVDNSISDEPSYRRVVE-OH	5005.45	MS^1	MS^2
MS-PP2$^{4-18}$	DVSPLAERVVNPNA-OH	1480.78	MS^2	MS^2
**Natalisin (Nat)**				
NAT-1	SPGQDEFGPFWANR-NH$_2$	1606.74	MS^1	MS^2
**Neuropeptide F2 (NPF2)**				
NPF2	ASSLCNRPLYSFETSKHVGDYLKCIANEASKTRY-NH$_2$	3850.90	MS^1	-
**Orcokinin-like (OK-like)**				
OK-like	LSPSKYETKTDKQ-OH	1524.79	-	MS^2
OK-like	GPLNGLIPGGAF-NH$_2$	1111.63	-	MS^2

**Pigment dispersing factor (PDF)**				
PDF	NSEVSNAIIGSEETQKLYRD-NH2	2252.12	MS^1	MS^2
**Proctolin**				
Proctolin 1	RYLPT-OH	649.36	MS^2	MS^2
Proctolin 1-PP2	SNSDRVDKLKELLKDLLESEIEKEEYQADAPPRWHPE SKLFY-OH	5045.55	MS^1	-
Proctolin-PP2$^{28-42}$	DAPPRWHPESKLFY-OH	1742.86	MS^1	-
**Pyrokinins (PK)**				
tPK	VSQNDFHHRHVNNQGNTHGGHIKEPYVWPSPKL-NH_2	3828.90	MS^2	MS^2
PK-1	HVVKFTPRL-NH_2	1095.68	MS^2	MS^2
PK-2	SPPFAPRL-NH_2	883.51	MS^2	MS^2
PK-3	HLPYLPRL-OH	1008.60	MS^2	MS^2
PK-PP3	HLPYLPRLRQNDRMPFS-OH	2140.12	MS^2	-
**Ryamide (RYa)**				
RYa1	VQNLSTFKTMMRY-NH_2	1617.82	MS^1	-
**Short neuropeptide F (sNPF)**				
sNPF	SSRSPSLRLRF-NH_2	1304.75	MS^2	-
sNPF-1(4-11)	SPSLRLRF-NH_2	974.59	MS^1	-
sNPF-PP2	SDPSMTPEAAFMMAQAVDHENN-OH	2392.98	MS^1	-
**SIFamide (SIFa)**				
SIFa	TYRKPPFNGSIF-NH_2	1425.76	MS^2	MS^2
**Sulfakinin (SK)**				
SK-1[pQ]	QTSDDYGHLRF-NH_2	1320.60	-	MS^2
SK-1[pQ]	QTSDDY(SO_3)GHLRF-NH_2	1400.55	-	MS^2
SK-2	GEETFDDYGHMRF-NH_2	1602.66	-	MS^2
SK-2 sulf.	GEETFDDY(SO_3)GHMRF-NH_2	1682.62	-	MS^2
**Tachykinin-related peptides (TKRP)**				
TKRP-1	APSGFTGVR-NH_2	890.48	MS^1	MS^2
TKRP-2 (x3)	APSGFMGMR-NH_2	952.45	MS^2	MS^2
TKRP-3	APMGFVGMR-NH_2	964.49	MS^2	MS^2
TKRP-4	APSGFLGMR-NH_2	934.49	MS^2	MS^2
TKRP-5	MPRQSGFFGMR-NH_2	1312.64	MS^1	-
TKRP-6	YPYEFRGKFVGVR-NH_2	1616.87	MS^1	-
TKRP-PP1	SIPDSAYSTGNSESDSTSELKAVDLVSDVGAVD-OH	3315.52	-	MS^2
TKRP-PP2	PYPVWEGTYPDGVY-OH	1642.74	-	MS^2
**NEUROPEPTIDE-LIKE**				
**Agatoxin-like (ALP)**				
ALP_b	ACVRRGGNCDHRPNDCCYNSSCRCNLWGSNCRCQRMG LFQKW-NH_2	4888.01	MS^1	-
ALP-PP (sulf.)	GPY(SO_3)LEDDEGLPSDDDYTENAIDRLLQSAQ-OH	3319.41	-	MS^2
ALP-PP	GPYLEDDEGLPSDDDYTENAIDRLLQSAQ-OH	3239.45	MS^1	-
**Neuropeptide-like precursor 1 (NPLP1)**				
	SISSLAQWGNLP-NH_2	1271.67	-	MS^2
	SLEALARAGYFRTLPADDDEDPNY-OH	2699.26	-	MS^2
	SLATLAKNGQLPTFQNNES-OH	2033.03	-	MS^2
	GIESLARNGELHT-OH	1396.72	-	MS^2
	NIGSLARNFNFPSY-NH_2	1598.81	MS^1	MS^2
	YLGSLMRGGDFQYT-NH_2	1606.77	MS^2	MS^2
	NIASLAREGGRFV-NH_2	1388.78	MS^1	MS^2
	NAAALLRQDNYLNAQRNEDKAEDSQAGG-NH_2	3031.46		MS^2
	NIASIKAQYSGKF-OH	1426.76	-	MS^2
**NVP-like peptide (NVP)**				
	IPASLVEEIKASEMRDNKV-OH	2129.13	MS^2	MS^2
	AQLSNGEEHDRADVPYFNKPTAI-OH	2572.24	MS^2	MS^2
	DARKIRLDGRF-OH	1346.77	MS^2	MS^2
	GRWGGFADA-OH	936.43	MS^1	MS^2
	GRWGGFA-OH	750.37	MS^2	MS^2

**Periplaneta neuropeptide-like precursor (Pea-NPLP)**				
	TFYPRLGLDSIGL-OH	1451.79	MS^2	MS^2
	SSSFEPPHSEVTHEH-OH	1706.74	MS^1	MS^2
	GSSKEEIADVISDKPLQIQ-OH	2057.08	-	MS^2
	SEDELDNEWLIERYHKAIAL-OH	2444.21	MS^2	-
	NAEYPLQSFKNHDQPA-OH	1858.87	MS^1	MS^2
**PROTEIN HORMONES**				
**Insulin like peptide_ILP1 transcript 1 (ILP)**				
ILP-PP1	SPRMVHLMN-OH	1084.54	MS^2	MS^2
ILP	EVYYCGSKLASALALVCNGKYNSPS-OH	2635.24	-	MS^2
**Ion transport peptide (ITP)**				
ITP-PP	SPANRSPALLPHHFT-OH	1644.86	MS^2	MS^2
**ITG-like**				
ITG-PP	LTGLATFKRPMH-OH	1371.76	MS^1	MS^2
**Prothoracicotropic hormone (PTTH)**				
PTTH-PP2	RFQDIDELPLKSNIND-OH	1916.97	MS^2	MS^2

*[M+Na]$^+$

¥[M+K]$^+$

Supporting information S1: List of neuropeptide precursors, neuropeptide-like precursors and protein hormone precursors from *Tenebrio molitor* and *Zophobas atratus*. Blue, signal peptide; amino acids in bold predicted possible cleavage sites of signal peptide; yellow, predicted sequence of bioactive neuropeptide; green, predicted C-terminal glycine amidation site; red, predicted cleavage sites of neuropeptides; light grey, predicted C-bridge site; red letters, amino acids substitutions between alleles. If different alleles or transcripts are listed, confirmation of identical sequences by mass spectrometry is included only in the first precursor sequence.

MASS SPECTROMETRY MS^2
MASS SPECTROMETRY MS^1
MASS SPECTROMETRY MS^2, AFTER TRYPSIN DIGESTION

Predicted *Tenebrio molitor* neuropeptide and neurohormone precursors

>*T. molitor Adipokinetic hormone-1*
MYRVLLIFLLVAFVGVCS**A**QLNFSPNWGKRVSSTTGGGESDNCKESMDVIMLIYKLIQNEAQKLVECEKFSN

>*T. molitor Adipokinetic hormone / corazonin-related peptide*
MIALLLLVTWTFINGAQ**A**QVTFSRDWNPGKRVAENTDFHNTMKTASAVCHLLINQVRQLATCDNNRGDDLDSGTP
TIFNGRR

>*T. molitor Allatostatin C* (= PISCF; AstCCC Veenstra2019)
MSAQVPHYLFRTLLVLFVATLAVSS**A**R**P**NHFGDPNQVVAEGDANNLLDSGLKPWQLEMLAQRLSEISQTGGDYGW
DKSIRSPESKRQSRYRQCYFNPISCFRK

>*T. molitor Allatostatin CC*
MNRILMLVLESFLVAVLFGSGTD**A**FVVDRRSAASERNSDDYPDYQLGVKYDEYPMIVPKKRTALLVDRLMVALQQ
AIEEEEAANRVDGPPLTDSFSLSPEEVRKMDLQRRGHGSMSGQQKGRVYWRCYFNAVTCF

>*T. molitor Allatotropin*
MAFQHAALFLTLIFLWLMLSNVQ**G**RREKVTQVRTPQQRLTRGIEHFKYHNMDLGTARGYGKRAVDMHNVNNFLLE
WIALETRMRDLGIPRNLLRDQETIPE

>*T. molitor Antidiuretic factor b-1*
MNSKISIILLLSLVAVAR**A**GVV**A**VPSVEVLQGPSSKTTIVGPDGSAISSVAPGGTVVTDGQAVVPAAPVVLAAAP
ATLVAGPAGSISTTHTLAGPAVVAAAPVAVAPVVVAGVEGHEGEYIPDNTEQLYDDGSYKGE

>*T. molitor Calcitonin_1*
MKAAFVLLAIALPAAY**C**FYLQPNYHVPARLGTRNAPVYKSLGDLFHRLHLASKRCVNTVDESCINGGGNDAGNDE
DFLNGGDTPGKRCANLYDESCSNGGINGAGADDDWLHGGNNPGRR

>*T. molitor Calcitonin_2* (Complemented with JABDTM020022981.1)
MKLSLLIFAVALSTAC**G**LYMYPKHRPRDEEVFQSLASFFNQLGRRANMKRCANTFDESCLNGPIGGATSDENWLS
NGSPGKRCSNIFGSSCVDGGTAGAGADEDFLGGGGGPGRR

>*T. molitor Calcitonin-like diuretic hormone* transcript a (= DH31 transcript 1 Veenstra 2019)
MKPSMIHSNGVSLLVLLAAGIILFEATTTY**A**APHSSRYYPGYYSPLSMEGQNPEYLLQTIARLRQALIADDDLEN
SKRGLDLGLGRGFSGSQAAKHLMGLAAANFAGGPGRRRRSEEEA

>*T. molitor Capa* transcript a
MMKTFLACSVHLCFVLFCVAVCL**A**E**S**KEPKRSKLSSVYALTPSLRVGRRSDDSWDPNTGRQPSGAAPAHFARLAD
VKRRIGKMVSFPRIGRGDSNWVADENNYGAKRPGANSGMWFGPRLGRLQKRNVDEFTPWTYIILNGEGPVSRQVH
YTPRLGRESDEVYDELDADVDVLA

>*T. molitor Capa* transcript b
MMKTFLACSVHLCFVLFCVAVCL**A**E**S**KEPKRSKLSSVYALTPSLRVGRRSDDSWDPNT----------------
DVKRRIGKMVSFPRIGRGDSNWVADENNYGAKRPGANSGMWFGPRLGRLQKRNVDEFTPWTYIILNGEGPVSRQV
HYTPRLGRESDEVYDELDADVDVLA

>*T. molitor CCHamide* 1
MCHKQTTMMSPLPVKLAKITVVVIFFCFAECAAGSCLSYGHACWGAHGKRNGAHNNNMPGRDAPPVSRDSTWFLS
KLVQSPLDLRYVNDKDLDLPTSQQLFADAQIEADPLKGQEDYRGLPDAYSNEENVLFDIYPNQRPRPNKIKASKY
LEKRSTRMI

>*T. molitor CCHamide* 2
MNCWSSVVVLLAVMAFVLAFHAEAAEAKRGCATFGHSCYGGMGKRASELMENNEEILQDVQGEENPAFVFTGPRS
EYKPERPPKLSPQQYDTISRVIRQWIQSYRGAQEMREN

>*T. molitor CNMamide*
MRIAFGVIFVTGIFGGFFGDNASASPVHHHHVISKDLDDTTADKMNKPYISISHDRADNSDKADVITRVQKVFVD
SPKSNVSKNGKQQKTKQTAYLLVKTMRQRNKRYISYLTLCHFKICNMGRKRTTRYFHMIRRLDNES

>*T. molitor Corticotropin-releasing factor-like diuretic hormone* (CRF-DH37) (= DH37-47_transcript-1 and -2 Veenstra2019)
MRVPVYLVCAALVVAVKSEDHNYGRLLEPIDVAADQETVSYLLPKLTAKYRPNNEWSSVTDPRFYVLTEMESNDI
DNQMPSERSIQRRSPTISITAPIDVLRKTWEQERARKQMVKNREFLNSLN

>*T. molitor Corticotropin-releasing factor-like diuretic hormone* (CRF-DH47)_ (= DH37-47 transcript-3 Veenstra2019)
MRVPVYLVCAALVVAVKSEDHNYGRLLEPIDVAADQETVSYLLPKLTAKYRPNNEWSSVTDPRFYVLTEMESNDI
DNQNKLGPELRSKRAGALGESGASLSIVNSLDVLRNRLLLEIARKKAKEGANRNRQILLSLGKRAFLQSRASGTY
DNNV

>*T. molitor Crustacean cardioactive peptide*
MTTATFFVICIAAVLTVETRSLFLPKSLGQNLAARERVVEPKKRPFCNAFTGCGRKRSNLPALTDQGEIIDESLG
SLLELSAEPAVEDLSRQIMSEAKLWEAIQEANMELHRRRQESAESAEDDVAVPPRSAAASCALPPCYI

>*T. molitor Ecdysis triggering hormone*
MRCYGILTVVVLFYSLLHNSLGDSNYFLKAAKNVPRIGRSNSKNTNIDEMGKFFMKASKSVPRIGRRNENLDYGQ
TVDKRDQLPGWSDIADRFEYEPELFTSPEILDHLEMGDDPSAYDWDKVRAKRDNKKHPRFQYLM

>*T. molitor Elevenin*
MGPAIVKDHQITLLLFVGVLFLSTLQMSSGSAIKGPDNEKKSQFSLTSYCRKHILSPPCRGHQKRDVLSRLGEDY
RLSALENDFSGVDRSGDFGAILKSPTLLGELLRKAVEASTEYDSDNGNYLN

>*T. molitor FMRFamide related peptides* (= FaRPs)
MLSLPIIITIFLVRVTWAYNEELYSLSENLDPYSYPSEYQEDSDFDVRRRNNNNFLRFGRSGGTNYDVEYDDFNE
DFARPTRSGKTEKNDHFIRFGRSKQDFLRFGRDQHRVVRDRSGNYLRFGRSVPNTEEKVRNKRDTYSEFKRGGSN
FMRFGRNSNFLRFGRNNEMTATSDPEKVQQLQESPLVQLLSELLEHIKKGQDKNRIV

>*T. molitor Hansolin*
MWRIIMTSLLYVVVIESRPMELLEGPLWLQDESISPAELFLRRNPDVLSQRSLHQNMYNPDTEYDLILPRSLANK
RAITMFSRWSPLSSIGKQRTPIRSNPNTLPFNQIRDRQHGQPLRWG

>*T. molitor IDL-containing*
MVRVAFSPHPLLLVTIVMAVCATIPHAVMAIDLSRLYGHVNSKRNGDACHPYEPFKCPGDGNCISIQYLCDGAPD
CPDGYDEDSRLCTAAKRPPVEETGSFLKSLLASHGPNYLEKLFGNKARDALKPLGGVDKVAIALSESQTIEDFGA
ALHLMRSDLEHLRSVFMAVENGDLGMLKSLGIKDSELGDVKFFLEKLVNTGFLD

>*T. molitor Inotocin (= Vasopressin-like)*
MSKLVVSIVLLAFIESLVSGCLITNCPRGGKRSKFGLAETSVKPCVSCGPGQSGQCFGPSICCGPFGCLLGTPET
LRCQREGFFHEREPCIAGSAPCRKNTGRCAFDGICCSQDSCHADKTCTSDEKSRAFPETLDLYSLLNYQGELGGD
K

>*T. molitor Insect parathyroid hormone*
MRTVAVCVVFVVMVMSVQNVFAGPRYRIKRVSDAHLADLQTRIALNNKLKGISITMPVGGRIDPLRIGRRRRSQ
SRFLDVLFNHSEEDKGDPVDFNDYESLIQRFRNLE

>T. molitor Myoinhibitory peptide (= allatostatin B)
MKDAVAAVAAKMLGLVLLACCLQASITRALSDETPMKSTNDNPQMDDEMSKRDWNKDLHIWGKRGWNNLHEGWGR
KRSAPSWGDQPAMEKRAWQNLHSGWGKRFAPEDEYAIRQLAAMLEPQYDEYNPEGDLDVNDDEKRNWGQFHGGWG
KRSKWDNFRGSWGKREPAWSNLKGIWGKRSVQDQIAQ

>T. molitor Myosuppressin
MHHYTLVAVAFAAVAALLTSSATATAISCPPNPLEASPYVRHLCYAIEQAISENAITDDQYRRVEERNVNGNAKR
QDVDHVFLRFGRRLGL

>T. molitor Natalisin
MLVSLKWLLLLSLTGVHAQEPRKRNSNGRFSFEEPILLEQNDDDVSCSIGSCVKRSGQDEFGPFWANRGKKDPTY
TRNKLFAEEPHWILVSREDQNQNEPFYVARGKKNKNNPFSRVNPFWKTLFDENWSKRQNVLQNLADLPIFIEQDR
KGRGKNAKNTDPQ

>T. molitor Neuropeptide F1 transcript a
MRWSALWWFAVVAAVVVLEGKWTLAAPSPRNDDMFKELLRLDQMYSSIARPRFGKRVETNSNFAPIEYEGQYQSE
DVGDWLPVRR

>T. molitor Neuropeptide F1 transcript b
MRWSALWWFAVVAAVVVLEGKWTLAAPSPRNDDMFKELLRLDQMYSSIARPSVRSGPTQPDNMGPKVQRAIKMLT
LQHLDRLYADQARPRFGKRVETNSNFAPIEYEGQYQSEDVGDWLPVRR

>T. molitor Neuropeptide F2 (NPY)
MLSYKLLFFLVVVMALFMSVPCETRECNRPKNSFENIKHVHEYLECMKSQVSTRYGKRAHPLLLGRLRPDLYNDY
ENNLQQLYDVLYADN

>T. molitor Orcokinin-like transcript a2
MWFITPLFVVFLALGAIDAAPNLARLESNYNPYGEIQSVMGRKSARNFGVLQLGGGYGVAKRFSPSSNKYEMKTE
KHRRGPLNGLIPGGAFGRAARSNCARTNCQTFAYDKLIKILKGPMELKLEPYFSFDVESRNDSGDGQGMRGGQEN
MPTSIDAYYTPYQSKQFLEVE

>T. molitor Orcokinin-like transcript a1
MWFITPLFVVFLALGAIDAAPNLARLESNYNPYGEIQSVMGRKSARNFGVLQLGGGYGVAKRFSPSSNKYEMKTE
KHRRGPLNGLIPGGAFGRAARSNCARYFSFDVESRNDSGDGQGMRGGQENMPTSIDAYYTPYQSKQFLEVE

>T. molitor Orcokinin-like transcript b (Complemented with CAJRHG030000017.1)
MWFITPLFVVFLALGAIDAAPNLARLESNYNQIASISSLPKQRTSSILGIKRSLDGIGGGNLKGRSLSHWKLPHL
GRSLDGIDGGLIGRSLDGIGGGNLVGRSLHARGFDGIDGGLIGRSIDGIDGDLIGRSLDGIGGGNLVGRSMDGID
GDLIGRSLDGIDGGLIGRSLDGIGGGNLVGRSMDGIDGGLIGRSIDGIDGDLIGRSLDGIGGGNLVGRSMDGIDG
DLIGRSLDGIDGGLIGRSLDGIGGGNLVGRALHVRGFDGIDGGLIGRSIDGIDGDLIGRSLDGIGGGNLVGRGTD
GIDGDLIGRSLDGIDGGLIGRSLDGIGGGNLVGRALHVRGFDGIDGGLIGRSIDGIDGDLIGRSLDGIGGGNLVG
RSTDGIDGDLIGRSLDGIGGGNLVGRSLDRIGGGNLVGRSTDGIDGDLIGRSLARINKLNGRSV

> T. molitor Orcomyotropin-like partial transcriptome
…GDLIGRSLDGIGGGNLVGRSLDRIGGGNLVGRSTDGIDGDLIGRSLARTNKLNGRSV

>T. molitor Pigment dispersing factor
MRCTVVVALLALGVAVAPSQGYPSPGDDYRYLDRDYASPGAHQLASWIASQLRPKEYAPAPEVPILPYRLPLQGK
RNSEVSNAIMGSEETQKMYRDGKK

>T. molitor Proctolin 1
MFDRKFVFAVFLVVFATLALETRHTVEARYLPTRSNGDRVDKLRELLKDLLQSEIEKEEYQADAPQRWHPENKLF
YKREAPAH

> *T. molitor Proctolin 2* allele 1_
MFARKFFFTACLVLFVSLALETSHTVHARYLPTRSNGDRVDKLRELLRDLLEKELDDGYQGYVFSKWHPENKLSY
NK

> *T. molitor Proctolin 2* allele 2 (JABDTM020014046.1)
MFARKFFFSACLVLFVTLALERSRTVHARYLPTRSNGDRVDKLRELLRDMLEKELDDEYQGYVISRMHPENKLSY
NK

>*T. molitor Pyrokinin*
MERIILVNLAVLCVAILLSEVVLSVPHYGSHQVSVRRERNDDKKQSYMWFGPRLGRKKRNSNIDPYRNREREQLA
TLLDVIQDSPWAIVAVNGKRHVVNFTPRLGRESGEEFVSSAAEDRWLQDPEMSGEMLSQRSPPFAPRLGRHLSPF
SPRLGRENDKNLF

>*T. molitor RFLamide*
MGWYAVIFLLLIRYAVTTTGILHSISVPQNLVDEDIHQSIDKIGDEATIENINQYEDVRLEHLGRLLANVLLQPW
PKNISPILYVEDHSSESIPNEIPENEIVETEELNSIPFKRSRYYRKYPWKRQNSRYDAENRYLCQPTKEDVFRLL
VALHEARQGNRGQIVNFCNRRRPASAIFTNIRFLG

>*T. molitor RYamide*
MHARKVIVLLVYILTVLVSVAVTKRYTSDKRVQNLSTFKTMMRYGRAGPNPNEKESKVNIHPRADAFFLGPRYGK
RSSWSPNASLVYPVSTPLCGLDEDLSCAYTGISDLYRCTPRKGRESDFSTSSN

>*T. molitor Short neuropeptide F*
MQGYSAMKCLCAVTCIMIVVATVTSAAPSYADYDNNIRDLWEMLLQKDALEDKFGGHQMVRKSSRSPSLRLRFGR
RSDSSMSPEAAFMMAQAVDHDN

>*T. molitor SIFamide*
MQLGFAKFLTVCIVATFLASWLVMAEATYRKPPFNGSIFGKRGATNEYDSASKALSAMCEIASEACQTWFPTQEK

>*T. molitor Sulfakinin*
MGMKSVFTGIFIISSMYLLFIHQFHNASAAPGNVNNLDSHRPRGRPFTRLTPRTSSQYARIKPEPFNEFIVDDDD
LFELSKRQTSDDYGHLRFGKRGEEPFDDYGHMRFGRSGGDK

>*T. molitor Tachykinin-related peptide*
MHSTTITTAVVLATIYVVCTAEEHHKRAPSGFTGVRGKKSIPDSAYSTGNAESDATSELKADVVSDVGAVNKRAP
SGFMGMRGKKPYPVWEGTYPDEVYKRAPSGFMGMRGKKDMEFTNYGDEYDKRAPSGFMGMRGKKEYDSLSNQYDG
YFEKRAPMGFVGMRGKKEYDELMEEKRAPSGFFGMRGKKMPRQSGFFGMRGKKYPYEFRGKFVGVRGKKASDNPE
SDYYNNVDLNTLGQDLDLNQLMLLLTENDGASDIWNGNNEVGQYSQK

>*T. molitor Trissin*
MNKNLVVVLIVIAGVVWGEVQSCTSCGSECQSACGTRHFRTCCFNYLKKRNSDSLAMDPSLRLELWLAKSRNPYF
QQQRNFLDSSLEMPETVNHDHDITQ

NEUROPEPTIDE-LIKE

>*T. molitor Agatoxin-like peptide* transcript a
MKYTWLVLASCTVMVLAELLPGATAGPYLEDDEGLPSDDDYTENAIDRLLQSAQKRSSLIYLFRRACVRRGGNCD
HRPNDCCYNSSCRCNLWGSNCRCQRMGLFQKWG

>*T. molitor Agatoxin-like peptide* transcript b
MKYTWLVLASCTVMVLAELLPGATAGPYLEDDEGLPSDDDYTENAIDRLLQSAQKRACVRRGGNCDHRPNDCCYN
SSCRCNLWGSNCRCQRMGLFQKWG

>*T. molitor Neuropeptide-like precursor1*(all potential cleavage sites marked)
MAVFGAPKFLIGTGILMSALFFMVKSDETCDIEIENTLRTLLSPQEFPTIQQQALRKDLLRRFQEALEKANLDDD
MNYKRSLSSLAQWDNLPDKRNLEALARAGYVRTLPSQDDEDPNYKRSLATLAKNGQLPTYQNNDSKRGIESLARN
GELTTRREIQELLDELYNKRNVGSLARNFNFPTYGKRFLGSLVRNGDSQYSGKRNIASLAREGGRFVGKRNVAAM
LRQDNYLNGQKSNEKVEGPELDNEKRNLASIKAQYSGKFKRAVRSKRQTSYYDDEGGELPSPVYQNQNVDDYEEL
VKALTGIYPNTDKRFLGSVAKTGWFRPTSRYRSPEKRHIGALARLGWLPTLRNVRRFNRSGRSTRGEGCRETSSD
GQAEDDAVAENSLSLGDKRFLLQPAVDKILLRKIFMHPRTHPFLSDLS

>*T. molitor NVP-like* (= Baratin) (all potential cleavage sites marked)
MELRCSLKWATLASCMVLTLAIPASLIEEIKASELRNNKVKRAHPPMNNVEERSRDVPYYSKPTAIKRGTNSLKN
PTPDQQSLNEWEQEQSLYQSPEGLANLQSGLYNNADAPFDDKSVAEYEKGFRYGTNKEKLDEALENAVLKSELYG
DPAPLNQYRYYENDDRRRRRRRDARKIRPDNRIKREVDLTPEEILTILTLYENERQSNGYRPWSGEPDNDQNNNI

EEEENWLDAPVYPHAAGHTNDIGPSYLLDEKPFEKRGRWGGFADNRKKRFMVAKKRNDPTRELRYLNGPNKNDYY
TLSQLLSNQREPNVPLYHRLVL

> *T. molitor Periplaneta neuropeptide-like precursor* (all potential cleavage sites marked)
MGLKSFSSVGRLLLIIIGLTW**A**QEDSLRSALNAIDRRQKDLDFSKYDDENLGEYGYSLEGPDDLAFLSPSDYTTD
RELDKNPAIDRLLLDYLEDGSYYDSGRANHDDDVKKRISSSFRERLEEDKEQQLEELAQNYLASLENDREGNDND
DYSELIRELWEKYRNNPSIYNHPNLYLNNEIKKRAYYPRFGLDSVGLRKRNKYYDGDTLSDPPYLYNYNSRDDFY
NKYEDEDDEKDVDSSYYDWARANKHYKRERKYDSRQKTYNSVKRFPVSKRSSSFEPPRDSEITHEHKRSTTKKDA
VGKTDPKVAQDLSNIFGTASTEKPTTSKPEKPKTMKKEQVKDGKKSKSAKSEKNKSVSSKEEVSEAASDKPLQIQ
KKSIDWSDYFGLDRRKKSEDELDSEWLMERYHKAVGLTSKRNAEYPLQSFKNHDQPSKKESEGSPERKKDLEEVK
ISEMDAKLKNMEDTIIDDALKYTGAHEGTTDSQEIQDVKDRVISRLAAAYSLEKMRRALGEYKLSIAKERERLRQ
QQHKNSDDYDISEEKRVSVPRKQAIDEDREKIPEGDNNIKCSQGDEDCEEQNYRTPSEVLEQTAFEECPRVQRAC
NEVATILGHYARVFETACNMHQMCLLCSNNSWFAPTRQCNVLFLTKAFELCDGKQECQKEAQKSVRYLLDVNRSL
RLEPLGDCELACPDRR

PROTEIN HORMONES

>*T. molitor Bursicon alpha* (Complemented with JABDTM020028536.1)
…VPWKLWRLTLLFAVLSSM**C**LDPRLTSNIKAT**A**ASTTDECQVTPVIHVLQYPGCVPKPIPSFACIGRCASYIQVS
GSKIWQMERSCMCCQESGEREASVSLFCPKAKPGERKFIKVTTKAPLECMCRPCTGVEESAVIPQEIAGYADEGP
LSNHFLKSHSQ

>*T. molitor Bursicon_beta*
MYNKILLCLVYVSYVS**S**V**S**EISEETCETLMSDINLIKEEFDELGRLQRICNGEVAVNKCEGSCKSQVQPSVITPT
GFLKECYCCRESFLRERTITLTHCYDPDGVRLTTENANSMDVKLREPAECKCYKCGDFSR

>*T. molitor Eclosion hormone 1*
MACRTRNLLGAALFVLLTSAFLLTE**A**NPIGVCIRNCAQCKKMFGPYFEGQLCADACVKFKGKIIPDCEDIASIAP
FLNKFE

>*T. molitor Eclosion hormone 2_*
MGSFGLIIALLLIGIIENCIC**G**ASIPVCITNCVQCKQMFGPYFQGKACGDACITSNGNLMPDCNNAGTLGNFLKR
LY

>*T. molitor Glycoprotein hormone alpha 2*
MLACWLLFTVLSLSD**A**F**M**VTTVN**A**RDAWQKPGCHKVGHTRKISIPECVEFHMTTNACRGFCESWAVPSGPKATPT
QPVTSVGQCCNIMETEPVEARVLCVDGVRTLTFKSAVSCSCYHCKKD

>*T. molitor Glycoprotein hormone beta 5*
MLSVQVWILVCLSALVS**S**Q**S**IIE**A**GLEPLDASGTIECHRRMYTYRVTQTDDNGKQCWDTLSVMACWGRCDSNEIS
DWRFPYKKSNHPVCVHYGRNRSVVTLRHCEEGADPTTARYEYLEAAGCKCQQCSSSDTSCEGLRYRPQRSHPASL
GFRIN

>*T. molitor arthropod insulin-like growth factor transcript a* (= Insulin 5 Veenstra 2019)
MNVPKGWIKMLCLVAAIGQIS**A**NIDSKEYFCGKKLVRTLTELCSIYNNPTYARNRFRRQIVDECCRSPCTRRYLV
LYYCSEAKSSVVSLLNRTKPENSSKQEKTGRSEPSPSEERNSLTRIKKLRRMQSPRNMIHNPVPPAKLGHVEHSQ
RPFYVWKFSRVY--

>*T. molitor arthropod insulin-like growth factor transcript b* (= Insulin 3 Veenstra 2019)
MMNVPKGWIKMLCLVAAIGQIS**A**NID**S**KEYFCGKKLVRTLTELCSIYNNPTYARNRFRRQIVDECCRSPCTRRYL
VLYYCSEAKSSVVSLLNRTKPENSSKQEKTGRSEPSPSEERNSLT------------------------------
------------VGRTLGTNNVPFMLNPDPLAYRRRLTQGRRKCVCRKRRAKVQ

>*T. molitor Insulin like peptide*_1 (= Insulin 1 Veenstra2019)
MDQRLLLLFLLVNSISVW**S**S**P**HKLHSINKRAEFFCGSKLSEALYMVCKGSYNSPTKKSINDLFAYEYDYFPSESD
EDNQLDFPFLERDTANSFLPIRSRRRRAGIVNECCRNPCTLQHLSLYCGS

>*T. molitor Insulin like peptide* 2 (= Insulin 2 Veenstra 2019)
MDLQCVFVVVVATVLAALHTCT**A**EDVATIRGSQNNKKIYCGTRLSETLSAVCKGNYNTLNKKSDIYSLSKSNVWG
GRHSSDSYRPLDYPYRSKASASSLITTFRQRRRRGVFNECCEKPCSHEELSSYCGNSK

>*T. molitor Insulin like peptide* 3
...DNRVTFVFFLLNIIYVWCTPHMASFMNKREVFCGPKLSDALALVCRGNYNPPSKKSMNDLLTYNTYDELFPS
ENDDDLDFPFIQKEAANSFLPMRFARSRGVVDECCKKPCTYRHLTLYCG

> *T. molitor Insulin like peptide* 4 allele 1
MTCIIRLVALLFVVESCAGALKVPFWSDKRILCGRLLENAQFLVCRGVPPAFKRSSLGWNEWDGEKLQKEEQVGA
IDKKEYFAKWRKHECCDKGCTSGDLKSYC

> *T. molitor Insulin like peptide* 4 allele 2
MTCIIRLVALLFIVESCAGALKVPFWSDKRILCGRLLENAQFLVCRGVPPAFKRSSLGWNEWDGEKLEKEEQVGA
IDKKEYFAKWRKHECCDKGCTSSDLKSYC

>*T. molitor Relaxin* (Insulin like peptide)
MWFPVSTIAALCVLLDVSDTTRSDNELELVFRDRSQSDWEEAWHKEKYTRCRETLIKHLYWACEKDIYRLTRRSD
QNGFNNYINNVDEEFPWMAPKKAKRLLRFRRGVNRRAGSSITSECCKSSGCTWEEYAEYCPTNKRYTSYV

>*T. molitor Ion transport peptide-like* transcript a (= ITP-A Veenstra 2019)
MSYRSSIIVNTQAVWVCMTLAVIIQAVTSIPTNGSPVLLPHHFTKRSFFDIQCKGVYDKSIFAKLDSICEDCYNL
FREPQLHSLCRKNCFTTDYFKGCLETLQLSDEEAQIQLWIKQIRGAELGGLGPSVSPPNTS

>*T. molitor Ion transport peptide-like* transcript b (= ITP-B Veenstra 2019)
MSYRSSIIVNTQAVWVCMTLAVIIQAVTSIPTNGSPVLLPHHFTKRSFFDIQCKGVYDKSIFAKLDSICEDCYNL
FREPQLHSLCRSQCFSTKYFVGCVESLLLSEEMPNFRKMIEYLSK

>*T. molitor ITG-like*
MRALIILFMACLLGHKAHGWGGLFNRFSPEMLANMGYGGHGGFIQRTGEGDEGILEEYASEGDEEPCYGKRCTAN
EHCCPGSVCVDVDGVVGSCLFAYGRRVGELCRRDSDCESGLVCAEAEPGVSTRVCRPPVHQDKQYSEPCNMSSEC
DISRGLCCQLQRRHRQAPRKVCSYFKDPLVCIGPVASDQIKNTIQHTAGEKRLTGLASFKRPMH

>*T. molitor Neuroparsin*
MCPSYNFATIVLVLTITVIIFSDKGTTMSLPCRRCFTSDECNSPPPDFCPYGENKNYCGRRVCSKGPGEKCSNDQ
YAILGTCGEGMWCSNKDNRCHGCFIATMTCYE

>*T. molitor Prothoracicotropic hormone*
...KNLIIFLFLISTFFTLNKSMEIMKNRNYRLLDYDEMNNSEESKCVDNDLCRNTFGEQVKKEVQEDEESIFAS
DEDKKKTTRLAPHYHSTRPMPCSCGIDFRLLDLGHHYFPRYLHTGVCKSEICRGLYRCVERHYKVRVLKQRDPRS
PEIKTTMTLPDTLKGMWQPELVTVTVACECSL

Predicted *Z. atratus* neuropeptide and neurohormone precursors

>Z. atratus Adipokinetic hormone 1
MHRVWLTVLLIALVGICAAQLNFSPNWGKRATSSAGGENDNCKESVDTIMLIYKIIQNEAQKLVECEKFSN

>Z. atratus Adipokinetic hormone / corazonin-related peptide
MAFQIRIFSTIAVFLLVTWMFLSDAQAQVTFSRDWNPGKRGENPDFHNAMKTASAVCHLLINQVRQLATCDNRDE
IEPGANNIFGGRR

>Z. atratus Allatostatin C (= PISCF)
MSAQTPHYLLSTLLIFVIATLAFSSARPNHFGEGNQVVAEQDGNNLLDPGLKPWQLELLAQRLSEISSQTGGDYA
WDRSLRSPEAKRQSRYRQCYFNPISCFRK

>Z. atratus Allatostatin CC
MNRILMVVLESFLVAVLFETKTDGFLIDRRSAASERSDDYPDYQLGVKYDEYPMIVPKKRTALLVDRLMVALQQA
IEEEEAANRVDGPPLTDGYSLSPEEVRKMDLQRRGHGSMGGQQKGRVYWRCYFNAVTCF

>Z. atratus Allatotropin
MAFHHAALFSTLIFFLWLLLVSSAQGRRDSKYPQVRTPQQRLTRGIEHLKYHNMDLGTARGYGKRAVDMHNVNNF
LLEWIALETRMRNLGIPRNLVRDQDTIPE

>Z. atratus Antidiuretic factor b-1
MKIQVAAFLAVVLLAATDASHLAAALVGPGTHGTILQGPSSKTSLVGPDGSHIAGVAAGGTVVAPALHGGVVSAA
VAPGYVAAGVPAVVGVHAGAFLAPGSGHEGQYVHDYTEHLYDDGSYKGDYYEGH

>Z. atratus Antidiuretic factor b-2 fragmented
MHSLTAAVFFVAVAAASASGILGVHGGLGVLGGLGGVGVGLGGVGLGLGGVGVGHGIVDPIAIANTVNAGRVTDV
GAPAVLSGPAGSVVRAGVAAVPAAIAGPAVIAGHGIAAPGIIGGAGLIGAGAGILGHGGLIGSGLEGQWIPDINE
KLYDDGSYKPHVYGH

>Z. atratus Antidiuretic factor b-3 fragmented
MNTLCVAVFVALVAVAHGSGIGLGLGGLGSAAVIAGPAGTVTTGGLGGAIIGPAGIAGAGILGHGIGAPLVTSQA
AAQLALQLSVPAGSGLEGQWIPDVNEKLYDDGSYKPHLW

>Z. atratus Antidiuretic factor b-4
MNSKICILLLSFFVLAKAGLVAVPSVEVLQGPSSKTTLVGPDGSSISSVSPGGTVVTDGQAVVPAAPVVLAASPN
TVVAGPAGSVITSHTLAGPAVVPVAAPAVVAAAPLAVGPAVALVGEGHEGEYIPDNTEQLYDDGSYKGE

>Z. atratus Calcitonin 1
MKAAFILLAIALPAAYCFYLQPNVHYMPARVNSRSAPVYKRLGDMFHRLHLASRRCINTFDESCINDNINGAVAD
EGFVNGGGGPGKRCVNTFDESCANGDIDGAGTDDDWLNGGDTPGRR

>Z. atratus Calcitonin-like diuretic hormone (= DH31)
MKTSMIHGNGVSLLMLLAAGVILFEASTTYAAPHSSRYYPGYYSPLSMEGQNPEYLLQTIARLRQALIADDDLEN
SKRGLDLGLGRGFSGSQAAKHLMGLAAANFAGGPGRRRRSEEEA

>Z. atratus Capa
MDIMKTLSGVQLCFVLLCLAACMAECKEPKRSKLSSDFGLTPFLRSGRGSEHSWDANVDVKRKIGKMVSFPRIGR
SESWGADENNYGAKRPGANSGMWFGPRLGRVQKRSDEYTPWTYIIVNGEGPVTRQVHYTPRLGRESEEVYDDLDA
EDVA

>Z. atratus CCHamide 1
MCHKTTGVSPLPVKLAKITVIVIFFFFAECAAGSCLSYGHACWGAHGKRGGSSHTQQEPPNVNKDASLILAKLLQ
PIDLRYASDRDMQMPTNQQLFSDAQIEAELLKGQDDFRNLQDGYLNDENVLADDMYPLQRTHSRRVRATKALEKR
STRMI

>Z. atratus CCHamide 2
MNCWSAAVLLAAVAFVLAE**A**AE**A**KRGCATFGHSCYGGMGKRASDLLDNNEEILQDVQNDENPAFVFTGPRSEYKP
ERPPKLTPQQYDNISRVIRQWIQYYRRAQELHADNNI

>Z. atratus CNMamide
...LRFSLFQNDRASNNNEVDVLSRVQQVYIDTPTTTVGKNGKQPKATKAYLLVKTLRQRNKRYVSYLTLCHFKI
CNMGRKRTTRYFHMIRRQDDNES

>Z. atratus Corticotropin-releasing factor-like diuretic hormone (= DH37)
MRVPVYLVCAALVV**A**VK**S**EDRTNYYGGKFLEPVDVAADQETVSYLLPKLAAKYRPNSEWSGVTDPRFYVLTEMES
NDIDNQVPSERTIQRRSPTISIAAPIDVLRKTWEQERARKQMLKNREFLNSLH

>Z. atratus Corticotropin-releasing factor-like diuretic hormone (= DH47)
MRVPVYLVCAALVV**A**VK**S**EDRTNYYGGKFLEPVDVAADQETVSYLLPKLAAKYRPNSEWSGVTDPRFYVLTEMES
NDIDNQNKLSPEARSKRAGALGESGASLSIVNSLDVLRNRLLLEIARKKAKEGANRNRQILLSLGKRAFLQSRAS
GNYDNNV

>Z. atratus Crustacean cardioactive peptide
MTMVTFFVICIAAVLT**A**E**T**Q**S**LFLPKSVAQNLGARERVIEPKKRPFCNAFTGCGRKRSNLPPLPEQRELTDESIG
SLLELSAEPAIEDLSRQIMSEAKLWEAIQEANMELSRRRQESAESSEEDVAVPARSAAGSCALPPCYI

>Z. atratus ETH
…FTTVVFIHILLHVTSGEGNYFLKAAKNVPRIGRSNSNKNTNIDEMGKFFMKASKSVPRIGRRNENFEYGQGVEK
RDQ…

>Z. atratus Elevenin
…SNILNRNYCRNHIFSPRCRGQQKRDVMSRPSEEYRLPVVEDFSGIDRSREITAILNSPTLLGELLRKIVETPEY
ESDTSNYLK

>Z. atratus FMRFamide related peptides (=FaRPs)
MVPLALIITTCLIHLTW**S**Y**N**EELYPLSDPDSSYLYPEDVPDETEFEIHRRNNNNFLRFGRSGRKYDSEYEDYNED
FARPTRSGKIDKNNDFFIRFGRSKQDFLRFGRDQERPVRAKNEFHLRFGRSMEGSQDRRRSKRDTYPEYKRAGSN
FLRFGRNSNFLRFGRNNEMAATADPEKQESPLIVLLTELVATLKKEQMKN

>Z. atratus Hansolin
MWRIIMTSLLYVVMVE**S**R**P**MDPLDGPLWPQDVSISTLDLFPRRNPDVRLRSFHQNMYNPDTDFDLLVPQFPANKR
AITMFSRWSPISSFGKGKTPTRSNMLPHQIKDRHPGQPLRWG

>Z. atratus IDL-containing
MVRVAFSPHPLLLVTIVMAVCATFPH**A**VM**A**IDLSRLYGHISSKRNGDACHPYEPFKCPGDGNCISIQYLCDGAPD
CPDGYDEDSRLCTAAKRPPVEETGSFLKSLLASHGPNYLEKLFGNKARDALKPLGGVDKVAIALSESQTIEDFGA
ALHLMRSDLEHLRSVFMAVENGDLGMLKSLGIKDSELGDVKFFLEKLVNTGFLD

>Z. atratus Inotocin (Vasopressin-like)
MSKLATLIILLALSE**S**IVS**G**CLITNCPRGGKRSKLALSENTIKSCLNCGPGQTGQCFGPNICCGPFGCLLGTPET
LRCQRDGFFHEREPCIAGTSPCRKNTGRCAFDGICCSQDSCHSDKACASEEKSRSFSEVPLDLYNLINYQAELVN
DK

>Z. atratus Insect parathyroid hormone
MNTVTVCVFTLVLVMSVQNVF**A**GPRYRVKRVSDAHLADLQSRIALNNKIKGISITMPVGGGRIDPLRIGRRRRSQ
SRFLDVLFNQSEEDKGDPVELNDYDSLIQRLRNFE

>Z. atratus Myoinhibitory peptide
MRDAAVAVSARFLGAVLFVCCLQ**A**SL**T**V**A**L**S**DETPMKSSNDNPQMDYDMSKRDWNKDLHIWGKRGWNNLHDGWGR
KRSVPSWADQADKRAWQNLHSGWGKRFTPEDEDTLRQLVAMIDRVDPQYDEYDNDLEANDDDKRNWGQFHGGWGK
RSNWGNFRGSWGKREPAWSNLKGIWGKRSQDQIAQ

>Z. atratus Myosuppressin
MHQYTFAAVVFGVAAVFLSN**A**S**T**T**A**YMISCPPNDLLEASPSLRHLCYIVEKAVVDNSISDEPSYRRVVERDVSPL
AERVVNPNAKRQDVDHVFLRFGRRFGL*

>*Z. atratus Natalisin*

MLISIKWLLLLTLTGVHAQEPRKRASNDRFTYEEPIVLEQEDDVSCSGGGCVKRSPGQDEFGPFWANRGKKDPTY
SRSKLYAEEPHWILVRRDDKDVNDNEPFYVARGKKSFSESNSKNIAAQFWRTLFGVNSPRKLKYLQNLGVPYFLE
VGRKENGNERSADIQ

>*Z. atratus Neuropeptide F1* transcript a

MRWSALWWFAVIAAMVMLEANAAPSPRNDNMLQELLKLDQLYSSVARPRFGKRVETNSNFAPIEYEGQYQSEDVG
DWLPLRR

>*Z. atratus Neuropeptide F1* transcript b

MRWSALWWFAVIAAMVMLEANAAPSPRNDNMLQELLKLDQLYSSVARPSVRSGPTQPDSMGPKVQRAINMLRLQH
LDRLYGDPSRPRFGKRVETNSNFAPIEYEGQYQSEDVGDWLPLRR

>*Z. atratus Neuropeptide F2 (NPY)*

MSTCKLAILLTVLLAVHLISGSPASSLCNRPLYSFETSKHVGDYLKCIANEASKTRYGKRAPPALIGKLRPYVYD
YDLQQIQDLLYPEN

>*Z. atratus Orcokinin-like* transcript a

MWCTTPLFALLVTLVVIDAAPKLSDYETGHYPYPQVGAIMGRKPARTFGVLQLGGGYGVAKRLSPSKYETKTDKQ
RRGPLNGLIPGGAFGRAARSDCSRSRCRSFTYDKFIKYLRVPQEEKLHPYFSFDSAENRNDSDGGVKDGQEKMPI
SIDAYTPYQYRKLVESNKYI

>*Z. atratus Pigment dispersing factor*

MQKIATLFVLTIGLIINPVLGYPYAGDDYRYLDRDYASPGAHQLASWIASQLRPPKELIPPQEVPILPYRLPLQG
KRNSEVSNAIIGSEETQKLYRDGRK

>*Z. atratus Proctolin*

MFDRKFVFAVLFVIFATLALETRHTVEARYLPTRSNSDRVDKLKELLKDLLESEIEKEEYQADAPPRWHPESKLF
YKREVPSH

>*Z. atratus Pyrokinin*

...ERVVVVNWVVICVLILLLEYISSVSQNDFHHRHVNNQGNTHGGHIKEPYVWPSPKLGRKKRNSSSNDFYDNL
QKEGLAMLIMDALQNGPLSNGNAGKRHVVKFTPRLGRESEEEIVNGIRPDENQWPSDDSAISAEYLYQRSPPFAP
RLGRHLPYLPRLRQNDRMPFS

>*Z. atratus RFLamide*

MGWYAEVTFLLLVRYTASATELLHSNLPQSLNDEDVDPNLVNIGEDDLSFVNQYEDAKLEHLGKMLANVLIQPWP
RGLSPLVYVENAPPEYFQGDETVESDPVDVEENANIPFKRSRYYRKYPWKRQNSRYDAENRYLCQPSKEDVFRLL
VALHEARQGNRGQMISFCNRRRPASAIFTNIRFLG

>*Z. atratus RYamide*

MHARKVIVVLVYILTVLVSLAVAKRYSSEKRVQNLSTFKTMMRYGRAGPTSNQKENKVNIHPRADAFFLGPRYGK
RSSWSPNASLVYPVSSTPLCGLDEDLSCAYTGISDLYRCTPRKGHESEEFSTTSN

>*Z. atratus Short neuropeptide F*

MARYSTAMKCLCAVTCIMIMAATVTSAAPSYADYDNNIRDLLDIFLQKEALEDKYGPVHQLVRKSSRSPSLRLRF
GRRSDPSMTPEAAFMMAQAVDHENN

>*Z. atratus SIFamide*

MHFSFAKLLTICTLALILASWLVPSEATYRKPPFNGSIFGKRGATSEYDSTGKALSAMCEIASEACQAWFPAQEK

>*Z. atratus Sulfakinin*

MGMKSVFTGIFLLSSIYLLFIHQFHNASAAPGNVNNLEAHRTRGRPFARLGSRGSGQYARIKPEPFGEFIVDDDD
LFELSKRQTSDDYGHLRFGKRGEETFDDYGHMRFGRSGSE

>*Z. atratus Tachykinin-related peptide*

MHSTTITTAVVLATIYAACAAEEHHKRAPSGFTGVRGKKSIPDSAYSTGNSESDSTSELKAVDLVSDVGAVDKRA
PSGFMGMRGKKPYPVWEGTYPDGVYKRAPSGFMGMRGKKDMEFTNYADEYIKRAPSGFMGMRGKKDYESLSNQYE
GYFDKRAPMGFVGMRGKKDYDELVEDKRAPSGFLGMRGKKMPRQSGFFGMRGKKYPYEFRGKFVGVRGKKANDVS
GSEYYNNIDLNSLGQDLDINQLMLLLTESDGESDVWNGNNEIGQYSQK*

>Z. atratus Trissin
MNKNLVVVLIVIAGVVW**G**EVQ**S**CTSCGSECQSACGTRHFRTCCFNYLKKRNSDSLAMDPSLRLELWLAKSRNPYF
QQQRNFLDSSLEIPETVNHNEVTK

NEUROPEPTIDE-LIKE

>Z. atratus Agatoxin-like peptide transcript a
MKYTWLVLASCVVLVL**A**ELLPGTS**A**GPYLEDDEGLPSDDDYTENAIDRLLQSAQKRSSLIYLFRRACVRRGGNCD
HRPNDCCYNSSCRCNLWGSNCRCQRMGLFQKWG

>Z. atratus Agatoxin-like peptide transcript b
MKYTWLVLASCVVLVL**A**ELLPGTS**A**GPYLEDDEGLPSDDDYTENAIDRLLQSAQKRACVRRGGNCDHRPNDCCYN
SSCRCNLWGSNCRCQRMGLFQKWG

>Z. atratus Neuropeptide-like precursor1 (all potential cleavage sites marked)
MAVFGAPKFFFGTGILMFALFFMVN**S**DETCDIEIENTLKALLTPQEYPSMQQQALRKDLLRRFQEALDRTDDEDE
TNYKRSISSLAQWGNLPGKRSLEALARAGYFRTLPADDDEDPNYKRSLATLAKNGQLPTFQNNESKRGIESLARN
GELHTRREIQELLDELYTKRNIGSLARNFNFPSYGKRYLGSLMRGGDFQYTGKRNIASLAREGGRFVGKRNAAAL
LRQDNYLNAQRNEDKAEDSQAGGGKRNIASIKAQYSGKFKRSARNKRQASYFDTESGEYPAPVYQNQNVDDYEEL
MNALTEAYPNNDKRFLGKLQGVLLRRKLICGSEIEKRHIGALARLGWLPTLRNVRRFNRSGRSTSLEGCRETSAD
GQTEDDAISENALSLEDKRFLLQPAVDRILLRKMFHPRMRSFLSDMS

>Z. atratus NVP-like (all potential cleavage sites marked)
MELRWSIRWATLASCLVLTL**A**I**P**ASL**V**EEIK**A**SEMRDNKVKRAQLSNGEEHDRADVPYFNKPTAIKRGTNNLKNP
TPDQQTLSEWEQEQNMYQNPDSLANIQSSLYNSDTPYDDKSIAEYEKGFHYGTNKDKLDEALENAVLKSELYGDP
SPLNQYRYYNGEDRRRRRRRDARKIRLDGRFKRDIDLTPEEILTILTLYENERQSGYRPWPMEGEPQADEIEEEE
NWMDAPVYPHATGHNDVASGYMYDEKAFEKRGRWGGFADARKKRFMVAKKRNDPTRELRYLNGPNKNDYYTLSQL
LSNQREPNVPLYHRLVL

> *Z. atratus Periplaneta neuropeptide-like precursor* (all potential cleavage sites marked)
MGLKSFTSVGRLLLILIGLTL**A**QEDSLRSALNAIDRRQKDLSEFSRYDDDNLGEYGYSLDAPDDLSFLSPSDYPE
RDFDKNPALERLLLDYLEDGSYLDSSRADHDEDVKKRISSSFRERLEEDKERQLEELAQNVLANDDRDNDDDYGE
LIRELWEKYKNYPNLYNHPRVYFNNDNKKRTFYPRLGLDSIGLRKRNKYYEGDSLNDNPYLYTYKDNYYNKYQDD
DDDDKDAEPNYYESAKNKHFSRDRKFDYRTRSYNPNKRFPVSKRSSSFEPPHSEVTHEHKRSTTKKDCVDKTDPK
VAKDLSNIFGSGSTEKPTTSKPEKPKTMKKEHAKETKSKNKTKGDKNKDKPKKGSSKEEIADVISDKPLQIQKKS
INWSDYFGLDRRKKSEDELDNEWLIERYHKAIALKRNAEYPLQSFKNHDQPAKKESDKTDSEEVKISEMDTKLKN
MEDTIIDDALKYTGAHEGTTDSEEIQDVKDKVISRLAAAYSLEKMRQALGEYKLSMTKEKDRLKQQHKSDDYDIS
EEKRVSVPRKQAIDDEREKIPESDNNIKCSQGDEECEDQNYRTPSDVLEQAVFEECPRVQRACNEIATVLGHYAR
VFETACNMHQMCLLCSNNSWFAPTRQCNVLFLTKAFELCDGKEECQKEAHGSIRYLLDVNRSLRLEPLGECELAC
PDRR

PROTEIN HORMONES

>Z. atratus Bursicon alpha
MKPFENLEVYKLWKLLFACAVL**S**SM**C**MDPRLSSNIKAT**A**ASTTDECQVTPVIHVLQYPGCVPKPIPSFACIGRCA
SYIQVSGSKIWQMERSCMCCQESGEREASVSLFCPKAKPGERKFIKVTTKAPLECMCRPCTGVEESAVIPQEIAG
YADEGPLSNHFLKSHSQ

>Z. atratus Bursicon beta
...SKVNSAFLKQKTNFDNSIPEEFDELGRLQRVCNGEIAVNKCEGSCKSQVQPSVITPTGFLKVRDDFAFSLTQ
MCFFQECYCCRESFLRERTITLTHCYDPDGVRLTVENANSMDVKLREPAECKCYKCGDFSR

>Z. atratus Eclosion hormone 1
MDTRTRNFIRTVLLMFLTASVFLVVD**A**NPIGVCIRNCAQCKKMFGPYFEGQLCADACVKFKGKIIPDCEDIASIA
PFLSKFE

>Z. atratus Eclosion hormone 2
MGSPTFIIVLVLMGIIENYICAASIPVCITNCVQCKQMFGPYFQGRACGDACISSNGQLMPDCNNPRTLGNFLKR
LY

>Z. atratus Glycoprotein hormone alpha 2

MLACWLLFTVLSLSD**A**FLVTTVT**A**RDAWQKPGCHKVGHTRKISIPECVEFHMTTNACRGFCESWAVPSGPKATPT
QPVTSIGQCCNIMETEPVEARVLCVDGVRTLTFKSAVSCSCYHCKKD

>Z. atratus Glycoprotein hormone beta 5
MFSVQVWILVGLSALVS**C**Q**S**IIE**A**GLEPLDVSATIECHRRMYTFRVTQTDESGKQCWDTLSVMACWGRCDSNEIS
DWRFPYKKSNHPVCVHYGRNRSVVKLRHCEEGANPSAARYEYLEAAGCKCQQCSSSDTSCEGLRYRPQRSHPASL
GFRIN

>Z. atratus Insulin like peptide 1
MDKRVIFLFFLLNVVSVW**S**SPRMVHLMNKREVYYCGSKLASALALVCNGKYNSPSKKSFDDLLAYDEYNEFFPSE
TDEDTQFDFPFLQKGAANSLLPMRFRKSKGIVDECCRKPCTLKHLELYCA

>Z. atratus Insulin like peptide 2
MDLQYVFMVVTTVLATIH**S**VK**T**DEMSTLNNSDSKKIYCGKNLSQTLSAVCKGNYNTLNKKSDIENRGRAVESQRG
QDFPFRNRAIASSLITNFRPRRRRGVFNECCEKPCSYKELSSYCGSNRKR

>Z. atratus arthropod Insulin-like Growth Factor 2
MYRFHGDLKMNVPKAWMQILCLVMLFGQMQ**A**NID**S**KEYFCGKKLVRTLTELCSIYNNPTFGRNRIRRQIVEECCR
SQCSRRYLVQYYCQVAKSPIAKLLNGTPENASKVAHEKDSNNAPSDHSAPSPAEERNSLNRIKKMRRLQSARNMI
HHNPVPPANIGHVEHSQKPFYVWRFSRMY

>Z. atratus Relaxin
...FPVSTIAALCVLLDVSD**T**TR**P**ENDLELVFRDRSQSDWEEAWHKEKYTRCRETLIRHLYWACEKDIYRLTRRS
DQNSYNNYVKN...

>Z. atratus Ion transport peptide like transcript a
MNYRSSRRISTQAVWVYITFAVILQEIA**T**S**P**AN**R**SPALLPHHFTKRSFFDIQCKGVYDKSIFAKLDSICEDCYNL
FREPQLHNLCRKNCFTTDYFKGCVETLQLSDEEAQIQVWIKQLRGAELGGLGPSVSPQNTS

>Z. atratus Ion transport peptide like transcript b
MNYRSSRRISTQAVWVYITFAVILQEIA**T**S**P**AN**R**SPALLPHHFTKRSFFDIQCKGVYDKSIFAKLDSICEDCYNL
FREPQLHNLCRSECFSTKYFVGCVESLLLNEEMPKYRKMIEYLSK

>Z. atratus ITG-like
MRSLIILFMACLLGHKAH**A**W**G**GLFNRFSPEMLANMGYGGHGGFIQRTGEGDEGILEEYASEGDEEPCYGKRCTAN
EHCCPGSVCVDVDGVVGSCLFAYGRRVGELCRRDSDCESGLVCAEAEPGVSTRVCRPPVHQDKQYSEPCNMSSEC
DISRGLCCQLQRRHRQAPRKVCSYFKDPLVCIGPVASDQIKSTIQHTAGEKRLTGLATFKRPMH

>Z. atratus Neuroparsin
MLSFHNFITVVLALTISVIIC**S**DRGT**A**LYHLPCKLCASVEECNDDPPQLCVYGENRNACKRRVCSKGPGEKCGDK
FDILGTCGEGLWCSSKDNRCHGCYMPTMTCYPQD

>Z. atratus Prothoracicotropic hormone
MRHLSLALLLLIFAFLRVNK**S**METWKDKYYGFLDYGDFSNNYSDDKCADNEVCQSNFDELVKNKRRFQDIDELPL
KSNINDKKSGRVATYYHSSRPMSCSCGIDFRILDLGHQYYPRFLHTGVCKTDICGGMYRCLEKYYKVRVLKQRDP
RSPEIKTSVAFPDNLKGTWQPEMVNVTVACECSL

Figure S1: Splice forms in *Tenebrio molitor*. **A** CAPA: T 1-174 – CAPA transcript a, T 1-158 – CAPA transcript b. **B** Neuropeptide F1 (NPF-1): T 1-85 – NPF1 transcript a, T 1-123 – NPF1 transcript b. **C** Corticotropin-releasing factor-like diuretic hormone (CRF-DH): T 1-125 – CRF-DH37, T 1-154 – CRF-DH47. **D** Ion transport peptide-like (ITP): T 1-135 ITP transcript a and T 1-120 ITP transcript b. **E** Arthropod insulin-like growth factor (aIGF): T 1-135 aIGF transcript a and T 1-120 aIGF transcript b. **F** Agatoxin – like peptide (ALP): T 1-108 – ALP transcript a, T 1-99 – ALP transcript b. For gene structures see Veenstra[8, 52]. Precursors aligned using the MAFFT-L-INS-i algorithm[95] (dvtditr (amino acid) Version 7.299b alg=A, model=BLOSUM62, 1.53, -0.00, -0.00, noshift, amax=0.0) and pictured using Jalview v.2.11.20. Color intensities indicate percentage of identity among amino acids.

A

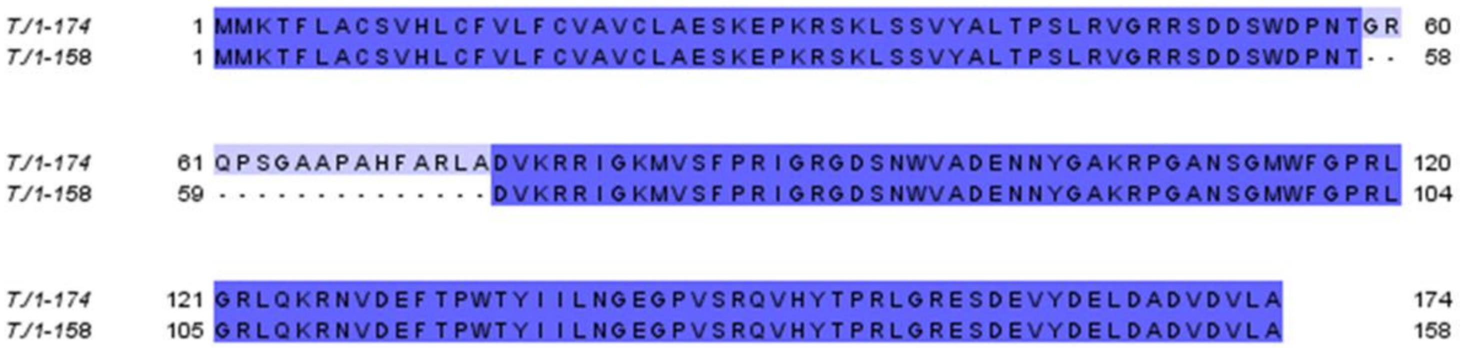

B

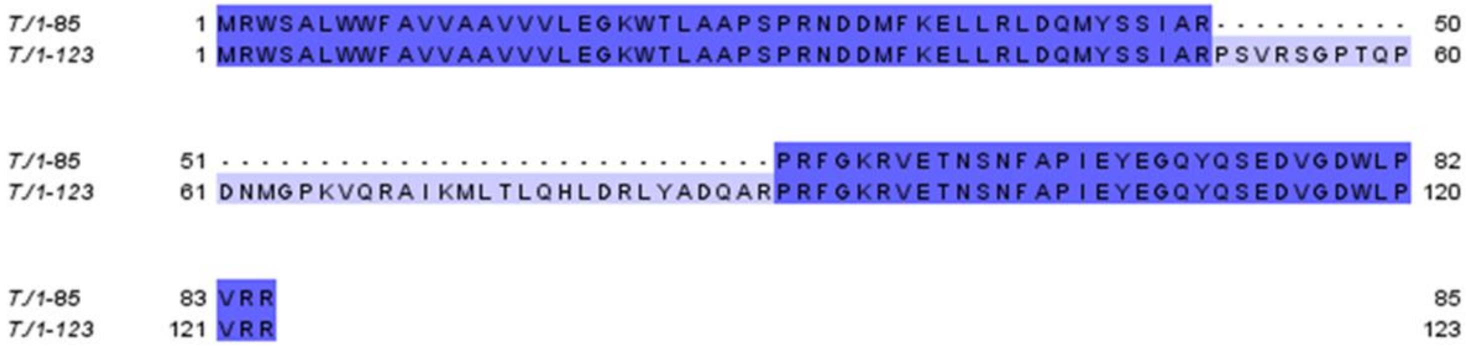

C

```
T./1-125    1 MRVPVYLVCAALVVAVKSEDHNYGRLLEPIDVAADQETVSYLLPKLTAKYRPNNEWSSVT  60
T./1-154    1 MRVPVYLVCAALVVAVKSEDHNYGRLLEPIDVAADQETVSYLLPKLTAKYRPNNEWSSVT  60

T./1-125   61 DPRFYVLTEMESNDIDNQMPSERSIQRRSPTISITAPIDVLRKTWEQERARKQMVKNREF 120
T./1-154   61 DPRFYVLTEMESNDIDNQ------------------------------------------  78

T./1-125  121 LNSLN------------------------------------------------------- 125
T./1-154   79 -----NKLGPELRSKRAGALGESGASLSIVNSLDVLRNRLLLEIARKKAKEGANRNRQIL 133

T./1-125      ---------------------
T./1-154  134 LSLGKRAFLQSRASGTYDNNV                                        154
```

D

```
T./1-135    1 MSYRSSIIVNTQAVWVCMTLAVIIQAVTSIPTNGSPVLLPHHFTKRSFFDIQCKGVYDKS  60
T./1-120    1 MSYRSSIIVNTQAVWVCMTLAVIIQAVTSIPTNGSPVLLPHHFTKRSFFDIQCKGVYDKS  60

T./1-135   61 IFAKLDSICEDCYNLFREPQLHSLCRKNCFTTDYFKGCLETLQLSDEEAQIQLWIKQIRG 120
T./1-120   61 IFAKLDSICEDCYNLFREPQLHSLCR----------------------------------  86

T./1-135  121 AELGGLGPSVSPPNT----------------------------------            135
T./1-120   87 ---------------SQCFSTKYFVGCVESLLLSEEMPNFRKMIEYLSK            120
```

E

```
T.J/1-162     1 MNVPKGWIKMLCLVAAIGQISANIDSKEYFCGKKLVRTLTELCSIYNNPTYARNRFRRQI  60
T.J/1-161     1 MNVPKGWIKMLCLVAAIGQISANIDSKEYFCGKKLVRTLTELCSIYNNPTYARNRFRRQI  60

T.J/1-162    61 VDECCRSPCTRRYLVLYYCSEAKSSVVSLLNRTKPENSSKQEKTGRSEPSPSEERNSLTR 120
T.J/1-161    61 VDECCRSPCTRRYLVLYYCSEAKSSVVSLLNRTKPENSSKQEKTGRSEPSPSEERNSLT- 119

T.J/1-162   121 IKKLRRMQSPRNMIHNPVPPAKLGHVEHSQRPFYVWKFSRVY------------------ 162
T.J/1-161   120 ------------------------------------------VGRTLGTNNVPFMLNPDP 137

T.J/1-162       ------------------------
T.J/1-161   138 LAYRRRLTQGRRKCVCRKRRAKVQ                                     161
```

F

```
T.J/1-108     1 MKYTWLVLASCTVMVLAELLPGATAGPYLEDDEGLPSDDDYTENAIDRLLQSAQKRSSLI  60
T.J/1-99      1 MKYTWLVLASCTVMVLAELLPGATAGPYLEDDEGLPSDDDYTENAIDRLLQSAQKR----  56

T.J/1-108    61 YLFRRACVRRGGNCDHRPNDCCYNSSCRCNLWGSNCRCQRMGLFQKWG             108
T.J/1-99     57 -----ACVRRGGNCDHRPNDCCYNSSCRCNLWGSNCRCQRMGLFQKWG             99
```

Figure S2. Orcokinin-like precursor alignments of *T. castaneum*, *T. molitor* and *Z. atratus*. A. Orcokinin-like transcript a: Tribolium 1-137 Orcokinin transcript a (Jiang et al., 2015); Tenebrio 1-171 Orcokinin-like transcript a2; Tenebrio 1-146 Orcokinin-like transcript a1; Zophobas 1-170 Orcokinin-like transcript. B. Orcokinin-like transcript b: Tribolium 1-184 Orcokinin transcript b (Jiang et al., 2015); Tenebrio 1-439 is deduced sequence by *T. molitor* genome scaffold CAJRHG030000017.1; Tenebrio 1-351 is deduced by *T. molitor* genome scaffold JABDTM020025471.1; and Tenebrio 1-57: is deduced by *T. molitor* transcriptome. For gene structure see Jiang et al., [64]. Orcokinin precursors aligned using the MAFFT-L-INS-i algorithm[95] (dvtditr (amino acid) Version 7.299b alg=A, model=BLOSUM62, 1.53, -0.00, -0.00, noshift, amax=0.0) and pictured using Jalview v.2.11.20. Color intensities indicate percentage of identity among amino acids.

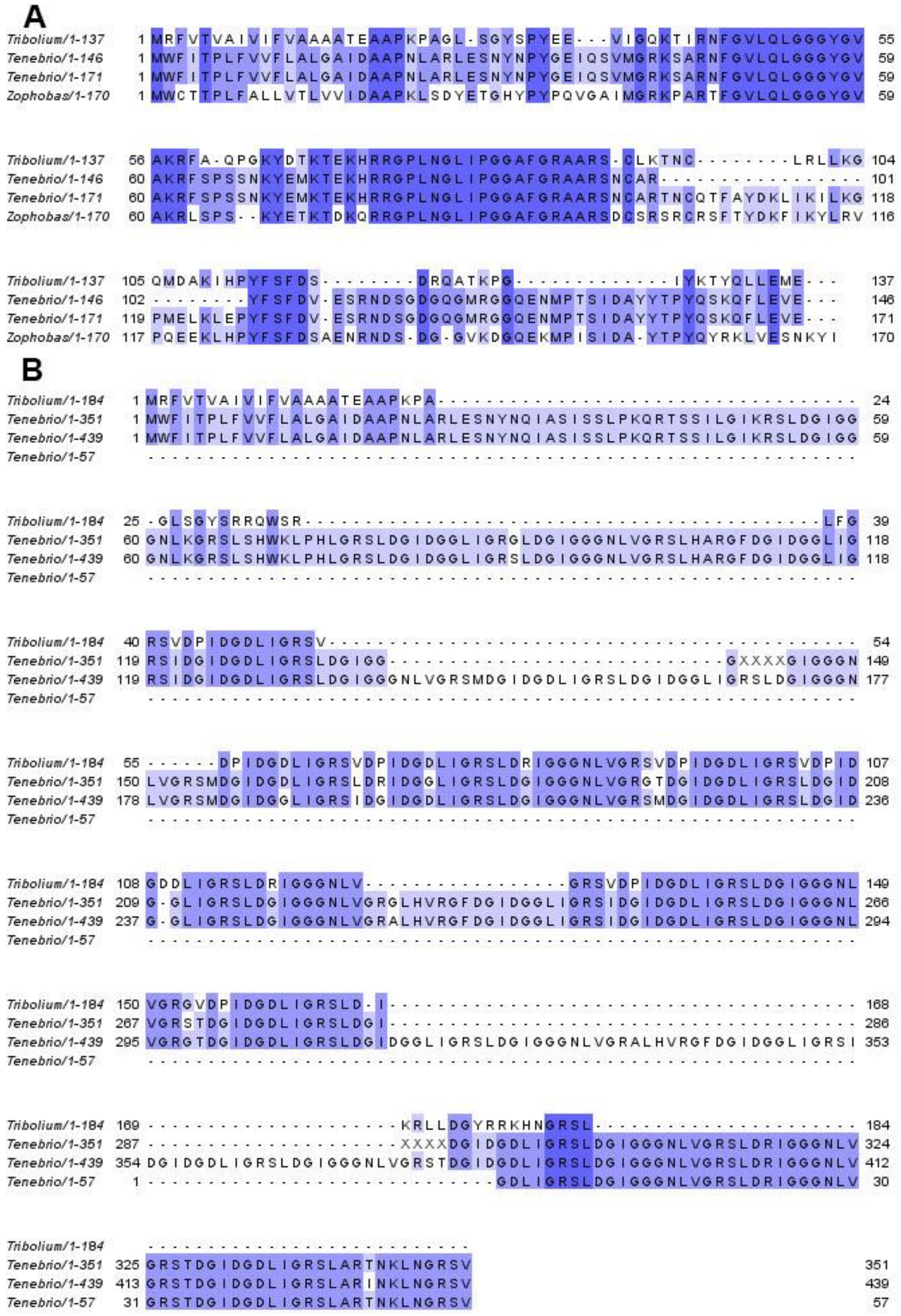

Figure S3: Proctolin 2 alleles of *T. molitor*. Ta1 1-77 – allele 1;, Ta2 1-77 – allele 2 (JABDTM020014046.1).

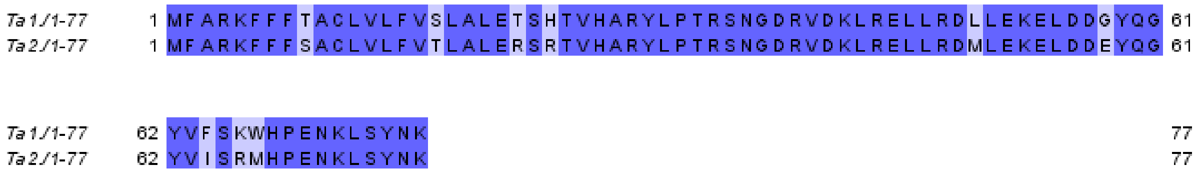

Figure S4: Splice forms in *Zophobas atratus*. **A** Agatoxin-like peptide (ALP): Z 1-108 – ALP transcript a, T 1-99 – ALP transcript b. **B** Neuropeptide F1 (NPF1): Z 1-82 – NPF1 transcript a, T 1-120 – NPF1 transcript b. **C** Ion transport peptide-like (ITP): T 1-136 – ITP transcript a, T 1-120 – ITP transcript b. Precursors aligned using the MAFFT-L-INS-i algorithm[95] (dvtditr (amino acid) Version 7.299b alg=A, model=BLOSUM62, 1.53, -0.00, -0.00, noshift, amax=0.0) and pictured using Jalview v.2.11.20. Color intensities indicate percentage of identity among amino acids.

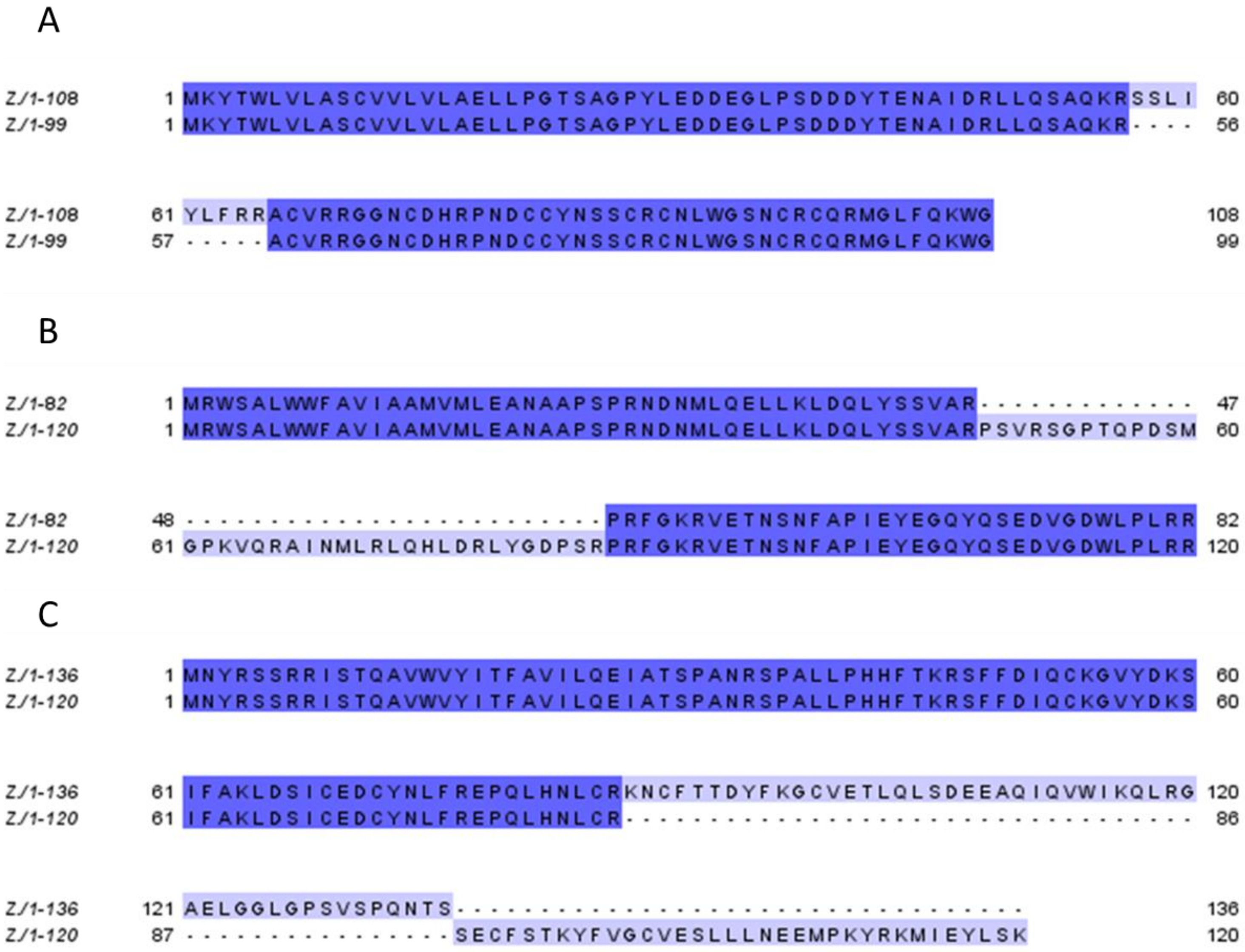

Supplement: Supplementary file 1 — pr1c00694_si_001.pdf [file pr1c00694_si_001.pdf]
